# Endoplasmic Reticulum‐Plasma Membrane Contact Sites as an Organizing Principle for Compartmentalized Calcium and cAMP Signaling

**DOI:** 10.3390/ijms22094703

**Published:** 2021-04-29

**Authors:** Tim Crul, József Maléth

**Affiliations:** 1 First Department of Medicine, University of Szeged, H6720 Szeged, Hungary; 2 HAS-USZ Momentum Epithelial Cell Signaling and Secretion Research Group, University of Szeged, H6720 Szeged, Hungary; 3 HCEMM-SZTE Molecular Gastroenterology Research Group, University of Szeged, H6720 Szeged, Hungary

**Keywords:** membrane contact sites, SOCE, STIM1, Orai1, calcium, cAMP

## Abstract

In eukaryotic cells, ultimate specificity in activation and action—for example, by means of second messengers—of the myriad of signaling cascades is primordial. In fact, versatile and ubiquitous second messengers, such as calcium (Ca^2+^) and cyclic adenosine monophosphate (cAMP), regulate multiple—sometimes opposite—cellular functions in a specific spatiotemporal manner. Cells achieve this through segregation of the initiators and modulators to specific plasma membrane (PM) subdomains, such as lipid rafts and caveolae, as well as by dynamic close contacts between the endoplasmic reticulum (ER) membrane and other intracellular organelles, including the PM. Especially, these membrane contact sites (MCSs) are currently receiving a lot of attention as their large influence on cell signaling regulation and cell physiology is increasingly appreciated. Depletion of ER Ca^2+^ stores activates ER membrane STIM proteins, which activate PM-residing Orai and TRPC Ca^2+^ channels at ER–PM contact sites. Within the MCS, Ca^2+^ fluxes relay to cAMP signaling through highly interconnected networks. However, the precise mechanisms of MCS formation and the influence of their dynamic lipid environment on their functional maintenance are not completely understood. The current review aims to provide an overview of our current understanding and to identify open questions of the field.

## 1. Introduction

Eukaryotic cells are characterized by a myriad of metabolic and signaling pathways in which pleiotropic second messengers—such as calcium (Ca^2+^) and cAMP—function as important connecting hubs between different cascades [1,2,3,4]. Although pleiotropic second messengers might generally offer flexibility to cells as to how to respond to certain stimuli, the logistic dilemma is, however, enormous and requires a strict spatiotemporal control in order to preserve the specificity of the intended cascade and avoid the development of pathological conditions [5,6,7,8].

Plasma membrane (PM) nanodomains—lipid rafts, caveolae, and membrane contact sites (MCSs)—serve as excellent tools allowing compartmentalization of initiators and modulators. For example, lipid rafts and caveolae are dynamic cholesterol-enriched PM microdomains, which play an important role in the initiation of many signaling pathways [9,10,11]. MCSs are dynamic close contacts between intracellular organelles with a major regulatory role on signaling cascades and cell physiology [12,13,14]. Affinity between the different membrane types as well as the maintenance of the MCS is mediated by a large variety of tether proteins, including, for example, extended synaptotagmins (E-Syts).

Next to being a general mediator of proper protein synthesis and transport, the endoplasmic reticulum (ER) acts as an important Ca^2+^ store [15]. Intracellular Ca^2+^ fluxes are, among others, generated through agonist-mediated release of cellular compartmentalized Ca^2+^ and subsequent activation of extracellular Ca^2+^ influx through plasma membrane (PM) channels through so-called store-operated Ca^2+^ entry (SOCE) within ER–PM contact sites.

Based on their biophysical properties, store-operated currents can be roughly divided into two different types: (a) the highly Ca^2+^ selective Ca^2+^ release-activated Ca^2+^ current (I_CRAC_), which is a non-voltage activated, inwardly rectifying current; and (b) a variety of store-operated currents classified as I_SOC_, which differ from I_CRAC_ in that, for instance, these currents are non-selective for Ca^2+^ and exhibit greater conductance compared to I_CRAC_ (reviewed in [16]).

Subsequent Ca^2+^-mediated activation of adenylyl cyclases within these MCSs allows specific spatiotemporal regulation of Ca^2+^-induced cAMP generation. Although a complete understanding of MCS functioning is still lacking, it is now clear that an intricate interplay between the MCS lipid environment and its resident proteins is crucial for it to function properly. Hence, considering that the activity of multiple ion channels and transporters is modulated by lipid species that are specifically enriched at ER–PM contact sites, this points towards a general role of the MCSs microenvironment in ion transport and cell physiology.

In the current review, the dynamics of ER–PM contact sites on SOCE-mediated cAMP signaling will be elaborated. Based on the current state-of-the-art, open questions will be identified that might enable the field to be pushed forward.

## 2. STIM/Orai/TRPC-Mediated Store-Operated Ca^2+^ Entry

Activation of G-protein-coupled receptors—seven-transmembrane receptors, including β2 adrenergic-like receptors activated by, for example, epinephrine; glucagon receptors activated by, for example, glucagon; and metabotropic neuroreceptors activated by, for example, small molecule neurotransmitters (such as dopamine or acetylcholine) or neuropeptides (such as encephalin [17])—results in synthesis of inositol triphosphate (IP_3_). Subsequent binding of IP_3_ to the inositol triphosphate receptor (IP3R) in the ER membrane induces Ca^2+^ efflux from the ER into the cytoplasmic space, enabling a myriad of cellular responses, including transcriptional regulation, exocytosis, and contraction.

Changes in ER luminal Ca^2+^ levels are monitored by single transmembrane-spanning stromal interaction molecule (STIM1 and -2) proteins and subsequently translated to the PM-residing Ca^2+^ uptake channels Orai and TRPC. Whereas Orai channels are highly selective for Ca^2+^, TRP channels are mostly non-selective cation channels permeable to both monovalent and divalent cations, including Ca^2+^ and Na^+^ [18,19].

At the protein level, STIM proteins contains an ER signal peptide, a canonical EF-hand Ca^2+^-binding motif as well as a hidden EF-hand, and a sterile alpha motif (SAM) at the N-terminal region, a transmembrane domain, and an ezrin/radixin/moesin domain, including three coiled-coil domains, a CRAC-modulatory domain, a proline/serine-rich region, and a polybasic lysine-rich region at the cytosolic C-terminus [20,21,22,23,24,25,26,27].

**Figure 1:**
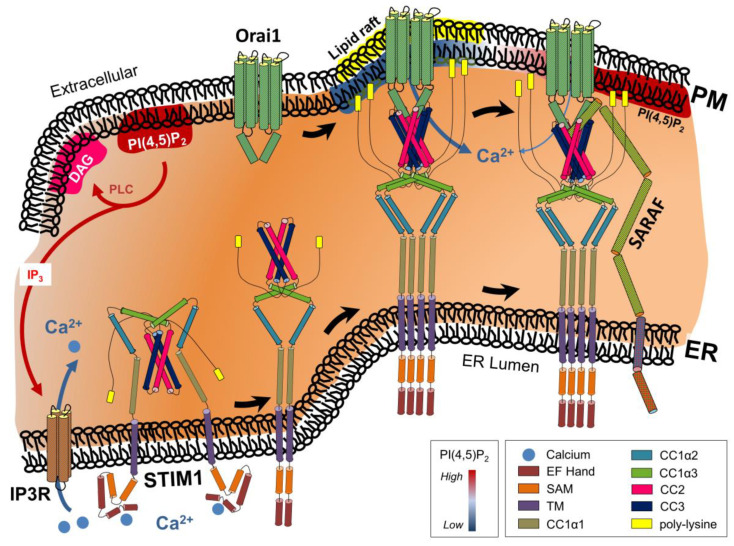
Receptor-induced activation of phospholipase C (PLC) generates inositol-triphosphate (IP_3_) and diacylglycerol (DAG) from phosphatidylinositol 4,5-bisphosphate (PI(4,5)P_2_). Binding of IP_3_ to its ER membrane-localized cognate receptor releases calcium (Ca^2+^) from ER into the cytosol. Subsequent depletion of the ER Ca^2+^ store induces conformational changes in the ER Ca^2+^ sensor stromal interaction molecule-1 (STIM1). Binding of STIM1 to the Ca^2+^ release-activated Ca^2+^ channel Orai1 within phosphatidylinositol 4,5-bisphosphate (PI(4,5)P_2_)-poor PM microdomains activates the channel, resulting in Ca^2+^ from the extracellular milieu into the cytosol. Interaction of store-operated Ca^2+^ entry-associated regulatory factor (SARAF) with STIM1 induces transition of the STIM1-Orai1 complex towards PI(4,5)P_2_-enriched PM microdomains, resulting in attenuation of Ca^2+^ influx. See the text for more details. Although the presence of cholesterol-enriched lipid rafts is needed for proper STIM1-Orai1 functioning, the precise interaction of lipid rafts and PI(4,5)P_2_ microdomains as depicted still needs to be clarified. Additional abbreviations: ER, endoplasmic reticulum; PM, plasma membrane.

STIM1-mediated sensing of the ER Ca^2+^ levels and subsequent activation of CRAC channels relies on extensive conformational changes of STIM1 (Figure 1).

At the luminal site, interaction of Ca^2+^ with the negatively charged aspartates and glutamates induces a helix-loop-helix conformation of the EF hand. Agonist-induced ER discharge and subsequent dissociation of Ca^2+^ from the STIM EF hand destabilizes this conformation, resulting in activation of STIM1 [28]. Interestingly, while the STIM1 TM domains are not in close proximity in resting conditions, the presence of three TM domain-localized glycine residues (G223, G225, and G226) aid in the movement of the STIM1 TM domains towards each other upon Ca^2+^ release from the luminal EF hand [29,30,31]. Subsequent homomerization of STIM1 is mediated by CC2, CC3, and SHD, which is essential for coupling to and activation of Orai1 [32,33]. X-ray crystallographic structural data of the STIM1 cytosolic region revealed a dimeric assembly with an antiparallel arrangement of CC2 and CC3 and two inter-adjacent short α-helices [34]. From these crystal structures, it could be derived that the physiological V-shaped conformation of the critical STIM-Orai1-activating region (SOAR) [26] (also referred to as channel-activating domain (CAD) [35], Orai-activating small fragment (OASF) [33,36], or coiled-coil domain-containing region b9 (CCb9) [37]) dimer is established through coiled-coil interactions of one monomer (residues R429, W430, I433, and L436) and the other (residues T354, L351, W350, and L347). Of note, mutation-induced disruption of this SOAR binding interface abrogated Orai1activation [34]. Even though only partial structural data is currently available of the STIM1 CC1 domain, this region comprises three alpha helical segments CC1α1 (aa238-271), CC1α2 (aa278-304), and the so-called inhibitory helix CC1α3 (aa308-337) [34,38,39]. While bridging the distance within ER–PM junctions mediating Orai1 interaction, the CC1 domain equally keeps STIM1 in a tight and compact conformation at high ER Ca^2+^ levels [29,36,39,40]. The polybasic cluster (K382, K384, K385, K386, and R387) located at the STIM1 C-terminal is—together with the SOAR domain—crucial for puncta formation through a diffusion-trap mechanism via interaction with PM-resident phospholipids and Orai channels; however, it does not mediate STIM1 oligomerization [32,35,41,42,43]. Overall, high ER Ca^2+^ levels preserve the STIM1 C-terminal region—including the CC1, SOAR, and polybasic region—in a folded (inactive) configuration. Depletion of the ER Ca^2+^ stores induces conformational changes of STIM1, resulting in extension of its C-terminal region, which exposes the SOAR and polybasic domain through the release of an intramolecular CC1-CC3 clamp, allowing interactions with PIP2 moieties in the inner leaflet of the PM (mediated by the polybasic region) as well as interaction and activation of CRAC channels (mediated by the SOAR region) [29,36,39,40].

In addition, STIM1 activity is regulated by a variety of post-translational modifications, including phosphorylation on serine [44] and tyrosine residues [45,46], and N-linked glycosylation at Asn_131_ and Asn_171_ located within the SAM domain [47].

Alternative splicing of STIM1 generates two splice variants: STIM1L, which is mainly expressed in skeletal muscle in human, and STIM1S, which has a more general expression profile [48]. Although both STIM1S and STIM1L interact with and activate Orai1 and TRPC, STIM1L does so with a much higher affinity and activating potential compared to STIM1S [49]. Alternative splicing of STIM2 gives rise to three splice variants: STIM2α/Stim2.2, which is able to interact with Orai1 and TRPC [44]; STIM2β/Stim2.1, a larger splicing variant with additional amino acids in the SOAR-region, which impairs its association with Orai and TRP channels [50,51]; and Stim2.3, a shorter variant lacking a lysine-rich region and the calmodulin (CaM)-binding domain at the cytosolic C-terminus (see further) [51]. ER store depletion-induced activation of STIM is modelled as a biphasic process [52]. Due to its lower affinity for Ca^2+^, STIM2 is more sensitive to changes in ER Ca^2+^ levels. Thus, the initial minimal Ca^2+^ depletion from the ER—approximately below 400 µM—is first sensed by STIM2 which—through triggering small and prolonged store-operated currents—corrects small fluctuations in ER Ca^2+^ levels [53]. Extended ER Ca^2+^ depletion activates STIM1, which initiates larger and transient store-operated currents to replenish the ER [41,52,53]. Physical interaction between both isoforms allows fine-tuning of the Ca^2+^ sensing process [44]. Moreover, heteromerization of STIM2.1 with STIM2.2 and STIM1 inhibits the function of the latter two, suggesting that variations in the expression ratio of these STIM variants might have a facilitative, redundant, or inhibitory effect on SOCE [50,51,54].

The three mammalian Orai subtypes—Orai1, -2, and -3—consist of a cytosolic N terminus followed by four transmembrane regions (TM1-4) and a cytosolic C-terminal extension (M4x). Crystallographic as well as functional studies revealed a hexameric arrangement of the Orai1 channel in which juxtaposition of the TM1 helices from all six Orai subunits form a highly selective Ca^2+^ pore [55,56,57,58]. Although the M4x region was originally identified as a STIM binding site [24,35,59], other Orai cytosolic domains—in particular, the N-terminus and intracellular TM2-TM3 loop—have been implicated [35,60,61,62,63], suggesting that multiple cytosolic domains may contribute to the binding with STIM. As such, by acting as a bridge between hexameric Orai channels, STIM facilitates the formation of a lattice structure consisting of STIM dimers crosslinking a multitude of Orai channel hexamers, implying the ability of STIM to ultimately coordinate oligomerization and hence control the density of Orai channels. Interestingly, liquid-phase electron microscopy indicated that a fraction of the present Orai1 channels form STIM-independent distinct supra-molecular clusters, suggesting that supra-molecular ORAI1 clusters might fulfill an amplifying function for creating dense ORAI1 accumulations upon SOCE activation [64]. Although initial studies indicated that association of STIM with both the N-terminal TM1-extension (M1x) and M4x region of Orai is essential for initiating the gating process [35,62,65,66,67], the current model proposes the M4x region as the prime interaction site for STIM. Next, interaction of STIM1 SOAR with Orai M4x induces a conformational change, which acts as a coupling trigger between the TM4 and TM3 helices to propagate structural alterations through the TM2 helix and ultimately pore-opening through conformational alterations of the neighboring TM1 pore helix [68,69,70,71].

TRPC channels are ubiquitously expressed and display diverse roles in most cell types [16,72,73]. When activated by receptor stimulation, TRPC channels function in STIM1-dependent or -independent modes. STIM1-mediated gating of the channels depends on specific TRPC multimer formation and consequently on the complement of TRPC channels present in the cells. In fact, when expressed alone, certain TRPCs (TRPC1, -2, -4, and -5) interact with STIM1, whereas others (TRPC3, -6, and -7) do not [32]. However, although characterized by a lack of interaction with STIM1 when expressed alone, TRPC3 and -6 do so when present in complex with the STIM1-interacting channels TRPC1 and -4 and consequently function as STIM1-dependent channels [74]. As such, the cellular composition as well as their ratio determines whether TRPC channels function in an STIM1-dependent or -independent manner. Interestingly, next to interaction with STIM1, parallel interaction of the STIM1/TRPC complex with Orai channels enhances the store dependence of TRPC [75,76,77]. Based on this, two mechanisms accounting for the observed channel interdependence are proposed: targeting of the channel to the PM from an intracellular pool and/or formation of STIM1/TRPC/Orai1 complexes at the ER–PM contact sites [78,79,80]. Direct gating of TRPC is regulated by STIM1 through interaction of positively charged lysines of STIM1 with negatively charged residues at the C-terminus of TRPC [35,81]. The precise mechanism of how such an interaction might result in channel opening is currently not entirely understood, although STIM1-mediated recruitment to PI(4,5)P_2_-rich domains has been proposed (see further).

Of note, spatiotemporal regulation of Orai1-STIM1 or STIM1-Orai1-TRPC1 ternary complex formation underlies the activation of different transcriptional programs driven by, for instance, NFAT (triggered by Orai1-STIM1+) or nuclear factor-kappaB (NF-κB) (triggered by STIM1-Orai1-TRPC1) [71].

Major regulation of STIM1 and STIM1-regulated Ca^2+^ entry is mediated through an interplay between the EF-hand domain family member B (EFHB) [82] and the single-pass type I membrane protein SARAF, which is expressed in the ER membrane [83] as well as in the PM [84]. Under basal conditions, E-Syt-dependent interaction of SARAF with SOAR maintains STIM1 in an inactive state and prevents STIM1–Orai communication [83].

Upon store depletion, STIM1 dissociates from SARAF and associates with the cytosolic Ca^2+^ sensor EFHB [82], forms STIM1-Orai1 complexes at ER–PM contact sites, and initiates SOCE [85]. Additionally, STIM1-independent interaction of SARAF with TRPC1 adjusts the Ca^2+^ influx and protects against Ca^2+^ overload [86]. By attenuating STIM1/Orai1-mediated Ca^2+^ entry, SARAF contributes to so-called slow Ca^2+^-dependent inhibition (SCDI) of Ca^2+^ influx through a destabilization of STIM1/Orai1 complexes [83,85]. Interestingly, SARAF-induced SCDI depends on the STIM1 CTID domain whereas Ca^2+^/calmodulin-induced SCDI does not, suggesting that both proteins operate through different mechanisms to attenuate Ca^2+^ influx [85,87]. On the contrary, STIM1-independent interaction of SARAF with Orai1 enhances Orai1-mediated Ca^2+^ entry [88]. These observations suggest that SARAF-mediated regulation of Orai1 and TRPC1 channels depends on the presence of STIM1. In cells co-expressing STIM1, SARAF regulates inactivation of SOCE [83,85]; in STIM1-deficient cells, SARAF rather enhances the Orai1 activity while attenuating TRPC1 function [88].

Next to attenuating STIM1/Orai complex-activity, increased [Ca^2+^]_i_ activates cytosolic Ca^2+^ clearance mechanisms [89] through which part of the Ca^2+^ re-enters the ER through ER-localized SERCA channels, while most of the Ca^2+^ is extruded out of the cytosol by PM-localized PMCA channels [90,91].

## 3. Ca^2+^ and cAMP Signaling Cascades form a Highly Interconnected Network

**Figure 2:**
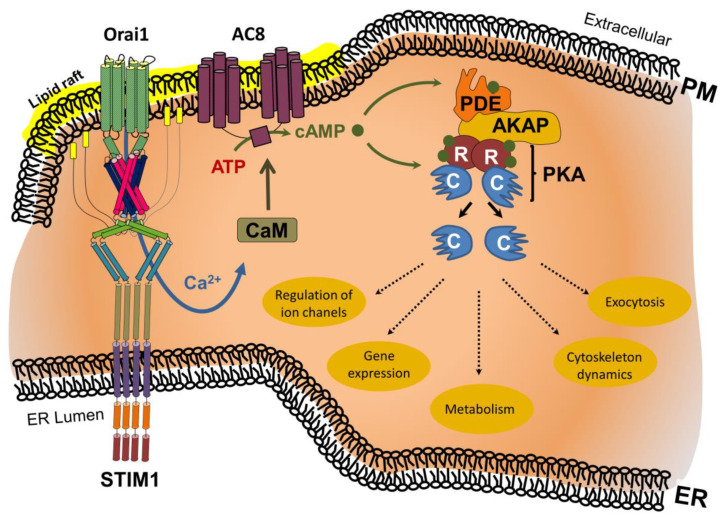
ER store depletion-induced Ca^2+^ influx triggers Ca^2+^/calmodulin (CaM)-mediated activation of adenylcyclase 8 (AC8) within lipid rafts. The resulting cAMP binds to the regulatory unit (R) of protein kinase A (PKA), resulting in the release of the PKA catalytic unit (C), and enabling regulation of ion channels, gene expression, metabolism, cytoskeleton dynamics, or exocytosis. Excess cAMP is broken down by phosphodiesterases (PDE). The range and selectivity of the cAMP-mediated biological effect is regulated by A-kinase anchoring protein (AKAP) through linkage of PKA and PDEs. Additional abbreviations: ER, endoplasmic reticulum; PM, plasma membrane.

Cyclic AMP (cAMP)—another highly abundant and versatile cellular second messenger—is generated from ATP through the activity of adenylyl cyclases (ACs) and metabolized by phosphodiesterases (PDEs). SOCE-induced Ca^2+^ influx directly modulates cAMP signaling cascades through Ca^2+^/calmodulin-mediated activation of Ca^2+^-dependent AC-5, -6, and -8 [92,93,94,95,96,97] (Figure 2).

Interestingly, although highly responsive to SOCE-mediated Ca^2+^ influx, these ACs are insensitive to alternative sources of cytosolic Ca^2+^ increase—for example, Ca^2+^ release from intracellular stores or those induced by diacylglycerol or arachidonate [93,96,97,98]—suggesting a close functional apposition of ACs with SOCE channels. In fact, even in the absence of SOCE, a close interaction of AC8 with Orai1 has been shown, suggesting stable complex formation between the AC and its regulator [99]. Of note, although direct interaction between AC8 and TRPC1 has not been evidenced yet, colocalization of AC8, TRPC1, and STIM1 was shown by TIRF analysis [100].

The efficiency and specificity of cAMP signaling is provided through A-kinase (PKA) anchoring protein (AKAP)-mediated multiprotein complex formation of cAMP signaling components. As such, AKAPs anchor the cAMP-regulated PKA in the vicinity of its substrates and ensure preferential phosphorylation of selected targets [101,102]. Next to PKA, AKAPs interact with regulatory proteins, including PDEs, protein kinase C (PKC), calcineurin, as well as all Ca^2+^-sensitive ACs, ultimately resulting in the establishment of cAMP microdomains [103]. For example, cytoplasmic retention of nuclear factor of activated T cells (NFATs)—due to extensive phosphorylation masking its nuclear localization sequence—is reversed by calcineurin-mediated dephosphorylation, enabling NFAT nuclear translocation and transcriptional induction of its responsive genes [104,105]. Upon store depletion, Ca^2+^ nanodomain formation near open store-operated Orai1 initiates AKAP79-mediated complex formation of calcineurin with Orai-tethered calmodulin, thus linking Ca^2+^ signaling with NFAT-mediated transcriptional regulation [106]. Of note, whereas knockdown of STIM2 expression had relatively little effect on Orai1/STIM1 clustering and subsequent Ca^2+^ influx, it significantly impaired NFAT1 activation and assembly of Orai1 with AKAP79, suggesting an important role for STIM2 in coupling Orai1-mediated influx to NFAT1 activation [107].

Obviously, the close interconnected character of both Ca^2+^- and cAMP-mediated signaling cascades provides multiple opportunities to mutually regulate the activity and outcome of each other. For example, Ca^2+^-mediated regulation of cAMP signaling at the level of ACs depends on AKAP-mediated compartmentalization of specific ACs in close proximity of Ca^2+^ transport proteins. In fact, the effect of Ca^2+^ on cAMP signaling is AC dependent as increased [Ca^2+^]_i_ results in activation of AC1, -3, and -8 but inhibition of AC5 and -6 [94,99]. Moreover, AC3 activity is indirectly attenuated through Ca^2+^/calmodulin protein kinase (CaMKII)-dependent phosphorylation of Ca^2+^/calmodulin [108]. In human melanocytes, binding of a-melanocyte-stimulating hormone (aMSH)—one of the major physiological determinants of melanogenesis—to G-coupled receptor melanocortin-1 receptor (MC1R) depletes Ca^2+^ ER stores and recruits STIM1 to ER–PM junctions. Next to Orai-mediated SOCE activation, STIM1 equally interacts with and activates AC6 at the PM, resulting in sustained elevated cAMP levels required to signal the induction of pigmentation genes [109]. Similarly, a crucial role of SOCE in fatty acid metabolism is evidenced by pathological amounts of lipid droplets (LDs) in skeletal and heart muscles of ORAI1- or STIM1/ STIM2-deficient mice or in isolated fibroblasts from human patients with loss-of-function mutations in STIM1 or ORAI1. In fact, SOCE regulates the expression of the neutral lipases hormone-sensitive lipase (HSL) and adipose triglyceride lipase as well as the cAMP-dependent activation of HSL and thereby controls lipolysis [110].

Of note, store-operated cAMP signaling (SOcAMPS) refers to the regulation of AC3 through ER Ca^2+^ depletion and subsequent clustering of STIM1 at PM Ca^2+^ microdomains [111]. Interestingly, this type of regulation is independent of Orai1, or elevation of cytosolic Ca^2+^ and could involve other ACs in a cell type-dependent manner [112,113]. For example, exposure of glucagon-releasing alpha cells to epinephrine stimulates sub-plasmalemmal translocation of STIM1 in a cAMP-dependent manner without formation of Orai1 protein clusters or SOCE, suggesting the involvement of additional factors in the formation of functional Orai1 channels and activation of Ca^2+^ entry [114]. Similarly, synaptic plasticity in excitatory neurons is mediated by cAMP-mediated migration of STIM2—but not STIM1—and the AMPA receptor (AMPAR) subunit GluA1 to ER–PM contact sites in dendritic spines. At these junctions, STIM2 promotes GluA1 phosphorylation though coupling of PKA to AMPAR in a SOCE-independent manner [115].

Next to modulating the activity of ACs, Ca^2+^ controls the extent of cAMP signaling through regulation of PDE activity. For example, while the affinity of Ca^2+^/calmodulin for PDEs is controlled by calmodulin kinase (CaMKII)-dependent phosphorylation [116], functional interaction of Ca^2+^/calmodulin with PDEs attenuates their auto-inhibition and increases their V_max_ [117,118,119]. Moreover, analysis of SOCE-induced modulations on PDE1 isoform activity suggests that fluctuations in [Ca^2+^]_i_ might facilitate the formation of intracellular cAMP domains [120].

In parallel, alterations in cAMP generation and subsequent changes in PKA activity equally regulate the magnitude and extent of [Ca^2+^]_i_ at multiple levels. For example, PKA-mediated phosphorylation of IP3Rs at multiple sites affects its activity in an isoform- and cell type-specific manner. In fact, whereas the output of IP3R1 is enhanced through PKA-mediated phosphorylation on Ser_1589_ and Ser_1755_ [121,122], neuronal IP3Rs are equally activated by phosphorylation of Ser_1755_ alone [123]. Moreover, PKA-mediated phosphorylation of IP3R2 Ser_937_ increases its activity in exocrine cells [124]. Interestingly, at submaximal IP_3_ concentrations, PKA-mediated phosphorylation enhances the open probability of IP3R1, suggesting a cAMP/PKA-mediated increased affinity of the IP3Rs for IP_3_ [121]. Combined with the observation that the affinity of IP3R1 for its inhibitor IRBIT is reduced through PKA-mediated phosphorylation [125], this indicates that PKA might regulate IP3R activity through sequential alterations in the affinity of IP3Rs for IP_3_ and IRBIT. Furthermore, AKAP79-mediated positioning of PKA in the proximity of Orai1 results in Orai1 Ser_34_ phosphorylation and subsequent Ca^2+^-dependent inactivation (CDI) [126]. While Orai1 channels exist as store-operated CRAC channels and store-independent arachidonic acid-activated ARC channels, CRAC channels are activated by ER membrane-localized STIM1 proteins whereas ARC channels are activated by a small PM-associated pool of STIM1 [127]. Interestingly, selective activation of ARC channels requires PKA-mediated phosphorylation of STIM1 Thr_389_, resulting in a conformational change of its SOAR region [127]. As such, PKA-mediated structural changes underlie the selective activation of STIM1-induced CRAC or ARC channels and determine the specific stimulation of these two functionally distinct Ca^2+^ entry pathways [128]. The cAMP/PKA pathway equally modulates [Ca^2+^]_i_ clearance. In fact, PKA-mediated phosphorylation of the SERCA inhibitor phospholamban (PLN) at Ser_16_ dissociates the PLN/SERCA complex and results in SERCA-mediated clearance of [Ca^2+^]_i_ into the ER [129]. Additionally, while calmodulin acts as the main activator of PMCA, PKA-mediated phosphorylation enhances the activity of PMCA in a Ca^2+^-dependent manner, suggesting that it might work through a Ca^2+^-dependent AC-mediated mechanism [130].

## 4. Compartmentalization of Ca^2+^/cAMP Signaling in Membrane Nanodomains at ER–PM Contact Sites

**Figure 3:**
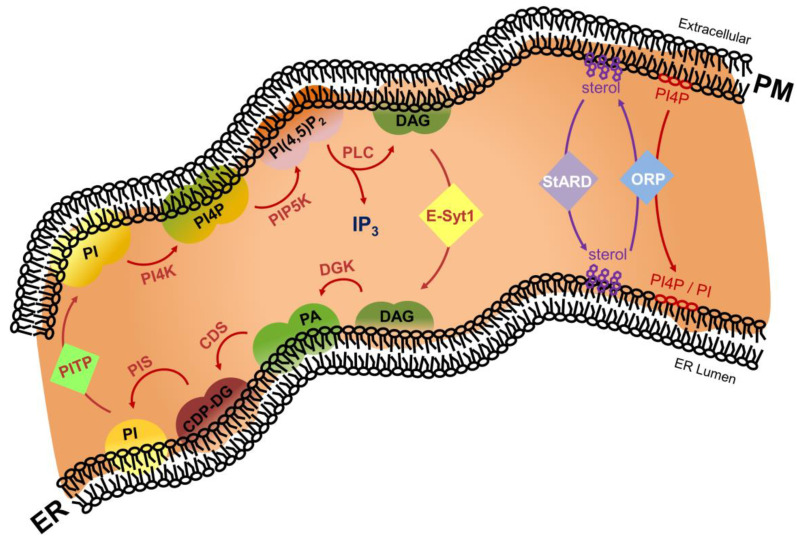
In the phosphoinositide cycle, phosphatidylinositol (PI) is sequentially converted to phosphatidylinositol 4-phosphate (PI4P) and phosphatidylinositol 4,5-bisphosphate (PI(4,5)P2) by PI kinase activities at the PM. Upon physiological triggers, phospholipase C (PLC) hydrolyzes PI(4,5)P2, generating the second messenger molecules diacylglycerol (DAG) and inositol trisphosphate (IP_3_). Extended synaptotagmin 1 (E-Syt1)-mediated non-vesicular transport of DAG from PM allows conversion of DAG into PI, PI4P, and PI(4,5)P2 at the ER [131]. PI4P species are transferred from the PM to the ER by oxysterol-binding protein-related protein (ORP). Cholesterol is cycled between the PM and the ER through the sequential actions of Star-related lipid transfer protein/Gram domain-containing protein (StARD/GRAMd) and ORP proteins. Additional abbreviations: ER, endoplasmic reticulum; PM, plasma membrane.

ER–PM contact sites are characterized by highly dynamic modulations of its lipid environment with a huge regulatory function on its resident proteins (Figure 3).

In fact, through spatiotemporal fluctuations of specific lipid species, ER–PM contact sites have a high degree of plasticity, which is a critical denominator of how they respond to physiological stimuli.

Through the so-called phosphoinositide cycle, phosphatidylinositol (PI) species in the PM are phosphorylated by phosphatidylinositol 4-kinase (PI4K) to generate phosphatidylinositol 4-phosphate (PI4P), which is subsequently phosphorylated by phosphatidylinositol 4-phophate 5-kinase (PIP5K) to generate phosphatidylinositol 4,5-bisphosphate (PI(4,5)P_2_). The latter is subsequently hydrolyzed to diacylglycerol (DAG) and inositol 1,4,5-trisphosphate (IP_3_) by phospholipase C (PLC). Next, DAG activates PKC whereas IP_3_ binds its cognate receptor in the ER membrane and activates Ca^2+^ release. Finally, upon E-Syt-mediated non-vesicular transport from PM to ER, DAG is converted to PI through the sequential action of diacylglycerol kinase (DGK), CDP-diacylglycerol synthase (CDS), and PI synthase (PIS). Finally, non-vesicular phosphatidylinositol transfer protein (PITP)-mediated transfer of PI from the ER to PM closes the circle, allowing additional rounds through the PI cycle. As such, the ER–PM contact site is an important regulator of Ca^2+^ signaling through spatiotemporal modulation of the levels of specific phosphoinositide lipid species.

In addition, the membranes that constitute the MCS are linked through protein–protein and protein–lipid interactions. MCS tethering proteins can be roughly subdivided into (i) structural proteins, tethers that hold the two organelles together; (ii) functional proteins facilitating the initially intended function of the MCS; (iii) regulatory proteins modulating the extent and function of the MCS; and (iv) recruitment/sorting proteins, which define the MCS-specific proteome and lipidome. Of note, this subdivision is most probably artificial as multiple proteins might in fact belong to multiple classes [132,133].

Extended-synaptotagmins (E-Syts) are ER-resident proteins characterized by a Synaptotagmin-like, mitochondrial and lipid-binding protein (SMP) domain. [134]. In mammalian cells, three E-Syts (E-Syt1, -2, and -3) are described [135]. In fact, through dimerization of their SMP domains, E-Syts function as homo- and heterodimers, which tether the ER to the PM through interaction with PM phospholipids, in particular PI(4,5)P2 [136,137]. Interestingly, PM interaction of E-Syt1 is enhanced by Ca^2+^, suggesting that [Ca^2+^]_i_ regulates the fraction of E-Syt1 present throughout the ER or concentrated at ER–PM contact sites [136,138,139]. Thus, considering heteromeric complex formation, both changes in [Ca^2+^]_i_ and PM PI(4,5)P2 levels regulate the formation/maintenance of E-Syt-dependent ER–PM contact sites [136,139,140]. Of note, although the rather high [Ca^2+^]_i_ concentrations—low micromolar Ca^2+^ range—required for E-Syt1 recruitment to the PM are established through activation of extracellular Ca^2+^ influx, including SOCE, E-Syts-dependent contacts are not required for SOCE itself [136].

At the MCS, E-Syts are essential for non-vesicular transport of DAG from PM to ER and for PI(4,5)P_2_ re-synthesis during repetitive rounds of PLC signaling [138]. However, interaction of E-Syts with other lipids was evidenced, suggesting their involvement in the transfer of as yet unidentified phospholipids [131,141]. Non-vesicular exchange of PI for phosphatidic acid (PA) between ER and PM is maintained by Nir2. PLC activation induces transfer of Nir2 into MCSs, where it interacts with vesicle-associated membrane protein (VAMP)-associated protein (VAP)—a tail-anchored ER membrane protein—and PA microdomains in the PM. Functional interaction between E-Syt1 and Nir2 has been suggested as E-Syt1 facilitates Nir2 recruitment and function at ER–PM contact sites [138]. Additionally, interaction of F-actin with Nir2 stabilizes the resulting ER–PM contact sites [142].

Next to agonist-induced modulations of the phosphoinositide cycle, the ER–PM contact site lipidome is equally characterized by alterations in PS and sterol levels. For example, VAP recruits ORP3 and ORP6 to ER–PM contact sites [143] which—together with the ER-embedded ORP5 and ORP8—mediate exchange of ER-derived PS and sterols for PM-localized PI4P [143,144,145,146,147]. Subsequently, PI4P is hydrolyzed by the MCS-residing Sac1 phosphatase [148]. Additionally, StARD/GRAM proteins extract accessible cholesterol from the cytoplasmic leaflet of the PM and transfer it to the ER [149]. Together, these lipidome alterations have a huge regulatory effect on the flux through the PI cycle. For example, ORP-induced lipid environment modulations through PS and sterol enrichments in the PM synergistically activate PIP5K and subsequent PI(4,5)P_2_ synthesis [143,150].

Through assembly into multimeric complexes and formation of linear filaments or other higher-order structures, septins operate as diffusion barriers and intracellular scaffolds during various cellular processes, including SOCE [151]. Loss of septin filaments dSEPT1 and dSEPT4 in Drosophila results in loss of the diffusion barrier and impaired dOrai activation by dSTIM, suggesting their role as positive regulators of SOCE [152,153]. Similarly, in human cells, SEPT4 promotes interaction between STIM1 and Orai1 through limiting the lateral mobility of Orai1 in the PM [154]. On the contrary, loss of dSEPT7 in Drosophila enhanced the intensity of dSTIM and resulting dSTIM-dOrai clusters near the ER–PM region [153]. Similar results were observed in SEPT7-knockdown human neurons differentiated from neural progenitor cells [155]. These observations suggest that—through uncoupling septin heteromers from ER-PM junctions—loss of SEPT7 influences the constitutive activation of Orai channels, thus allowing the STIM interaction with Orai [153,155].

The family of anoctamins—ER–PM contact site tethers homologous to yeast Ist2 [156,157]—consists of 10 members. Although some have been reported to function as Ca^2+^-activated Cl^−^ channels (ANO1 and ANO2) [158,159,160,161] or lipid scramblase and Cl^-^ channel (ANO6) [162,163,164], little is known about their cellular functions. Recently, ANO8 was reported to regulate multiple steps of ER depletion-induced Ca^2+^ signaling, including the formation of STIM1 dimers and puncta, STIM1–Orai1 interaction, SOCE, and SCDI [165]. Importantly, a crucial role for ANO8 in assembling the core Ca^2+^ signaling complex into PI(4,5)P2-rich domains at the ER–PM contact sites was evidenced [165].

As part of the cytoskeleton, microtubules—tubulin polymers—provide structure and shape to eukaryotic cells. Plus-ends of microtubules associate with end-binding proteins (EB1, -2, and -3), which in turn interact with STIM1 [166]. Complex formation between EB1/EB3 and STIM1 mediates ER movement and prevents excessive SOCE activation through sequestering STIM1 in the microtubules [167,168,169,170].

## 5. Regulatory Interplay between ER–PM Contact Site Lipid Species and Residing Proteins

### 5.1. ER–PM Contact Site-Residing Proteins Modulate Their Surrounding Lipid Environment

ER–PM contact sites are characterized by a dynamic spatiotemporal interplay of its residing proteins and its lipid microenvironment [171]. Not only are proteins attracted to and/or remain inside the ER–PM contact site based on their specific interactions with selected lipid microdomains, the lipid environment of ER–PM contact sites is equally modulated by ER–PM contact site-residing proteins (Table 1).

For example, upon association of E-Syts with the PM, micromolar [Ca^2+^]_i_ concentration stimulates E-Syts-mediated bidirectional transport of glycerolipids—including DAG—between ER and PM driven by the lipid concentration gradient in the membranes [141,217]. As such, E-Syts might be part of a homeostatic response needed to reset the PM lipid composition to normal levels after acute perturbations by transferring excess DAG from the PM to the ER for its metabolic recycling [218]. Additionally, ORP5 and -8 are recruited to ER–PM contact sites through interaction of their pleckstrin homology domain with PI(4,5)P2 pools in the PM, where it subsequently modulates PI(4,5)P2 levels [194]. Additionally, association of ORP3 with the PM is determined by both PI(4,5)P2 and PI4P. Upon activation, ORP3 extracts PI4P and PA from the PM while increasing its cholesterol and PS levels [150,190]. Similarly, the recruitment of ORP6 to ER–PM contact sites is involved in the turnover of PI4P [143,150]. Subsequently, clusters of PS and PI4P activate PIP5K, suggesting that ORP/Osh proteins create a PM lipid environment favoring PIP5K activity and PI(4,5)P2 synthesis [147,150]. Similarly, StARD/GRAM-induced cholesterol extraction from the PM might influence the organization and biophysical properties of the PM and thus the dynamic events taking place at the PM [131].

### 5.2. ER–PM Contact Site-Residing Lipid Species Modulate Protein Activity

Membrane composition is crucial in defining ion channel structure and function, either through specific interactions between lipids and proteins or nonspecific through changes in membrane physicochemical properties—thickness, fluidity, and curvature—that affect ion channel dynamics. Lipids directly interacting with membrane-embedded proteins are defined as annular lipids, those lipids in the first ring surrounding the protein, and non-annular lipids, lipids present in clefts or at the protein subunit interface. For example, molecular dynamics (MD) simulations emphasize the crucial influence of membrane structure and thickness on the function of membrane channels and provide important insights into the influence of the lipid microenvironment on protein function (see [219,220,221] for recent reviews). In fact, surrounding lipids directly affect stimulus-induced conformational changes necessary for efficient gating of channels while providing a specific/selective medium for small molecules to approach and interact with them.

MD simulations capturing cholesterol-induced modulation of protein structure and dynamics indicated a cholesterol-mediated effect through both specific binding interactions or through alterations in membrane bulk properties as well as through the formation of lipid domains with saturated phospholipids and sphingolipids [221,222,223,224].

Initial studies indicated that cholesterol depletion impairs interaction between STIM1 and both Orai1 and TRPC1 upon emptying of intracellular Ca^2+^ stores, suggesting their association with lipid raft domains [225]. Moreover, lipid raft domains were shown to be involved in the activation but not the maintenance of SOCE, probably due to the support of the formation of Ca^2+^ signaling complexes involving STIM1, Orai1, and TRPCs [226]. In fact, TRPC channels are characterized by a conserved Cav-1 binding domain allowing Cav-1-mediated targeting of TRPC proteins into lipid raft/caveolae domains [227]. Silencing of Cav-1 results in attenuation of SOCE and misallocation of TRPC1 and TRPC4 in airway smooth muscle and endothelial cells, respectively [228,229]. Interestingly, although TRPC1 interacts with Cav-1 [227,228,229,230,231], once inside the raft region, TRPC1 is regulated by STIM1 [232,233]. In fact, interaction of TRPC1 with STIM1 results in its integration into lipid raft domains, where it functions as a store-operated channel; in the absence of STIM1 interaction, TRPC1 functions as an agonist-activated channel outside rafts [232]. Interestingly, SOCE attenuates TRPC1-Cav1 complex formation in an STIM1-dependent manner [234], allowing interaction between TRPC1 and STIM1 in the lipid rafts [233]. Next to TRPC proteins, Orai1 also possesses a Cav-1-binding domain allowing Orai1-Cav-1 complex formation [235,236], suggesting a role for Cav-1 in the proper targeting of Orai1 into the lipid raft/caveolae regions [237]. Interestingly, the absence of Cav-1 results in impaired ER depletion-induced interaction of STIM1 with TRPC1 and microdomain localization in salivary gland cells of Cav-1-negative mice, although the interaction between STIM1 and Orai1 was unaffected [230]. Of note, interaction of Cav-1 with the STIM1-Orai1 complex selectively regulates the SOCE-induced activation of downstream NFAT- or c-fos-mediated transcriptional programs. In fact, phosphorylation of Cav-1 Tyr_14_ impairs c-fos activation without impacting the NFAT pathway or Orai1 activity indicating that—potentially through selective targeting towards specific PM microdomains—structurally distinct regions of Cav-1 selectively regulate the ability of local Ca^2+^ to activate distinct downstream transcriptional cascades [238].

In addition, cyclodextrin-induced cholesterol depletion attenuates SOCE in a wide variety of cells [226,233,239] accompanied with the dissociation of STIM1, Orai1, and TRPC1 [240]. Interestingly, whereas STIM1 puncta formation is absent in cyclodextrin-treated cells [233], overexpression of Orai1 and STIM1 is able to overrule the cyclodextrin-induced cholesterol depletion effect on SOCE [239]. However, lipid rafts are only essential during STIM1-Orai1-TRPC1 complex formation during initial activation of SOCE; when the complex is assembled, cyclodextrin-induced cholesterol depletion has no effect on SOCE nor on the complex itself [226]. However, recent studies suggest that the amount of cholesterol in the PM is monitored by the Orai1 amino terminus, which could modulate its activity during SOCE [241]. Whereas, under basal cholesterol conditions, most Orai1 channels are restricted in a confined space, reducing PM cholesterol induces Orai1 internalization and affects the lateral movement of Orai1, resulting in unobstructed diffusion in the plane of the PM. Since overexpression of Cav-1 during cholesterol depletion maintained Orai1 into a confined area and movement, this suggests a direct cholesterol-mediated regulatory effect on Orai1 localization and compartmentalization and SOCE [242]. Moreover, STIM1 possesses a cholesterol-binding motif inside the SOAR domain. Reduction of PM cholesterol levels detaches SOAR from PM while enhancing its association to Orai1 [243].

Anionic lipids, such as PI species, are asymmetrically distributed over the inner and outer leaflets of the PM by active transporters maintaining a higher concentration of these lipid species in the inner leaflet, a process of high physiological importance [244]. Due to its highly negatively charged nature, MD simulations evidenced, for example, PIP2 clustering around integral membrane proteins and its capacity to regulate their activity through interaction with both their transmembrane domain and their cytosolic linker/domain or juxtamembrane domain [219,220,221]. The lysine-rich polybasic motif located at the C-terminus of STIM1 allows interaction of STIM1 with anionic phospholipids, such as PIP2 and PIP3, in the PM [40,245,246]. Interestingly, whereas efficient binding of STIM1 to PIP2 requires tetramerization of its lysine-rich domain, association of STIM2 with PIP2 in contrast relies on dimerization of its lysine-rich domain, resulting in enhanced affinity for PIP2 and a lower activation threshold of STIM2 [247]. Upon deletion of this polybasic motif, ER depletion-induced translocation of STIM1 to the PM is impaired although STIM1 is still able to form oligomers in puncta [41]. Moreover, phosphatidyl kinase inhibitor-induced depletion of PI4P results in decreased Orai1-mediated Ca^2+^ entry although STIM1 puncta formation is preserved [248]. In fact, STIM1 first associates with PM-localized PIP2 and PIP3 pools in raft domains before its actual interaction with Orai1 [43], suggesting that cholesterol indirectly orchestrates proper STIM1-mediated targeting of SOCE components into lipid rafts through preservation of the required phosphoinositide pools. In addition, an N-terminal polybasic arginine-rich motif of Orai1 is involved in directing the channel to distinct PIP2 pools [249]. Interestingly, transient shuttling between PI(4,5)P2-rich and -poor domains is an important regulator of Ca^2+^ transport proteins and Ca^2+^ signaling. In fact, ER store depletion induces STIM1/Orai1 complex formation in PI(4,5)P2-poor regions. Due to the lack of PI(4,5)P2, SARAF is unable to interact with STIM1, thus allowing maximal Ca^2+^ influx. Next, the STIM1-Orai1 complex migrates into PI(4,5)P2-rich domains (or PI(4,5)P2 translocates to the STIM1-Orai1 complex), allowing SARAF to interact with STIM1 followed by SCDI-mediated Ca^2+^ influx attenuation [202]. Thus, rather than going through cycles of PI(4,5)P2 hydrolysis and re-synthesis, STIM1/SARAF-mediated regulation of Ca^2+^ influx through Orai1 operates by translocation between PI(4,5)P2 domains.

Additionally, TRPC channels are regulated by PI(4,5)P2 [250,251,252,253]. As such, although the STIM1-mediated gating of TRPC is not entirely understood, STIM1-mediated targeting of TRPC channels into PI(4,5)P2-rich domains could be essential for keeping the TRPC channels in an active state [254].

Ca^2+^-regulated adenylyl cyclases—AC1, -5, -6, and -8—are targeted to plasma membrane rafts, whereas Ca^2+^-independent adenylyl cyclases—AC2 and -7—are not [255]. Cyclodextrin-induced cholesterol depletion destroys these interactions and disrupts the regulation of the raft-localized ACs by SOCE, indicating that the presence of these complexes in raft domains is essential for their assembly and functioning [28]. The underlying mechanism targeting ACs to rafts is not completely understood. Although protein–protein interactions with the cytosolic region of AC5 and AC6 seemingly underlie their residence in rafts [47,48], the involvement of additional—weaker—protein–lipid interactions has been suggested [256]. In fact, a dynamic interaction between Cav-1 and the raft-targeting sequence located on the cytoplasmic domain of AC8 affects its processing, targeting, and responsiveness in plasma membrane lipid rafts in an N-glycosylation-specific manner [257,258]. Moreover, at the PM, AC8 enhances cortical actin and directly associates with cholesterol, suggesting that AC8 tracks along the cytoskeleton into cholesterol-enriched domains, where the produced cAMP modulates its association with the actin cytoskeleton. As such, AC8 actively orchestrates its microenvironment into a highly organized signaling hub [259]. The responsiveness of AC8 to SOCE is regulated through its direct interaction with AKAP79. Proper targeting of AKAP79 to lipid rafts is mediated by the association of three N-terminal polybasic regions with PM phospholipids as well as specific palmitoylation of specific cysteine residues, which regulate SOCE-dependent AC8 activity and subsequent PKA-mediated phosphorylation of raft-residing proteins, including AC8 [260]. Moreover, AKAP-mediated targeting of AC8 (and potentially other ACs) to PM raft regions enables direct interaction with Orai1, where they regulate the activity of each other [99].

## 6. Physiological Effects of ER–PM Contact Sites

Similar to Orai, additional ion transporters and channels are structurally and functionally modulated through alterations in their lipid environment [261,262,263]. Although currently understudied, the regulation of these transporters and channels by MCS-enriched lipid species strongly suggests their presence at MCSs. For example, arachidonic acid (AA) modulates the activity of the AA-regulated channels (ARCs), which are composed of STIM1-assembled Orai1/Orai3 heteromers [264]. As STIM1-mediated Orai activation occurs at ER–PM contact sites, ARC activation within this MCS is anticipated [131]. Additionally, whereas most TRPC channels are regulated by DAG, a clear connection with ER–PM contact sites is of yet not proven [122]. Furthermore, several channels, including K^+^, Ca^2+^, TRP, ANO1, and ENaC Na^+^ channels [265]; Na^+^/H^+^ exchangers [266]; Na^+^.HCO_3_^−^ cotransporters [267,268]; and PMCA pumps [269], are regulated by PI(4,5)P_2_ through, for example, direct interaction of PI(4,5)P_2_ with the transporter [270,271] or receptor-stimulated reduction in PM PI(4,5)P_2_ levels [272]. Together, these observations clearly suggest that the dynamic modulation of the ER–PM contact site lipid environment has a huge effect on transporter function, signaling, and cell physiology.

## 7. Conclusions and Outlook

It is clear that, through dynamic modulations of its lipid environment and associated proteins, the ER–PM contact site plays a huge regulatory role on the spatiotemporal activity of the versatile second messengers Ca^2+^ and cAMP. Therefore, a better understanding of this complex regulation may also reveal unknown aspects of crucial cell functions, such as ion and fluid secretion [273,274,275,276,277,278]. However, as we only start to appreciate its complexity, many open questions still need to be addressed. For example, identifying additional MCS-resident proteins and their function(s) might give clues on how the formation and dissolution of membrane contact sites is regulated. Additionally, a clear understanding of the interplay between lipid environment modulations and protein function within the MCS is still missing, mainly due to the highly dynamic character of these modulations and the lack of high-resolution fractionation methods as well as the complexity of lipidomic analyses. Finally, knowledge of how (disrupted) MCSs function in pathological states could provide insight in the underlying mechanism and offer new functional therapeutic interventions.

## Figures and Tables

**Table 1:** Overview of ER–PM contact site-residing proteins. Abbreviations: AKAP79, A-kinase Anchor Protein 79; ANO8, Anoctamine-8; CDS, CDP-Diacylglycerol Synthase; DGK, Diacylglycerol Kinase; E-Syt1, Extended Synaptotagmin-1; GRAMd2, GRAM domain-containing protein 2; IP3_R_, Inositol Triphosphate Receptor; Kv2, Delayed rectifier potassium channel; Nir2, Pyk2 N-terminal Domain-Interacting Receptor 2; Orai1, Calcium Release-Activated Calcium Channel Protein 1; ORP, Oxysterol-Binding Protein-Related Protein; PI4K, Phosphatidylinositol 4-kinase; PIP5K, Phosphatidylinositol 4-Phosphate 5-Kinase; Phosphatidylinositol Synthase; PKA, Protein Kinase A; PLC, Phospholipase C; Sac1, Phosphatidylinositol-3-Phosphatase; SARAF, Store-Operated Calcium Entry-Associated Regulatory Factor; SERCA, Sarcoplasmic/Endoplasmic Reticulum Calcium ATPase 3; STIM1, Stromal Interaction Molecule 1; TMEM24, Transmembrane Protein 24; TRPC1, Transient Receptor Potential Channel 1; VAP, Vesicle-Associated Membrane Protein-Associated Protein.

Proteome	Localization	Trigger	Interaction	Function
AKAP79	PM (PI(4,5)P_2_) [172]		PKA [101]	Regulates positioning of STIM1/STIM2/Orai1/PKA [107]
ANO8	ER [165] PM (PI(4,5)P_2_) [165]	PI(4,5)P_2_ [165]		Mediates Orai1 inactivation through Recruitment of ER-localized SERCA [165]
CDS	ER [173]			PA→CDP-DAG [173]
DGK	ER [174]			DAG→PA [174]
E-Syt1	ER [135]	Ca^2+^ [136]		Transfer of DAG and other uncharacterized lipid species [141]
	PM (PI(4,5)P2) [136]			Modulation of membrane curvature [175]
F-actin				Stabilizes Nir2-containing ER-PM MCS [142]
GRAMD2	PM (PI(4,5)P_2_) [176]			Sterol transfer (PM→ER) [177]
				Pre-form ER-PM MCS for STIM1-mediated SOCE [176]
IP_3_R	ER [2]	IP_3_ [2]		Outward-rectifying Ca^2+^ channel [2]
Kv2	ER (VAP) [178]	Phosphorylation [178]	VAP [178]	Delayed rectifier potassium channel [179]
Nir2	ER (VAP) [180]		VAP [180]	PI transfer ER→PM [181]
	PM (PA) [182]			PA transfer PM→ER [181]
Orai1	PM [183]		STIM1 [184,185,186]	PM-localized inward-rectifying Ca^2+^ channel [183]
ORP	ER (VAP) [187]		VAP [187]	Sterol, PS transfer (ER→PM) [188]
				PI4P→PI transfer (PM→ER) [188]
ORP3	ER (VAP) [189]	PKC [190]	VAP [189]	Sterol, PS transfer (ER→PM) [190]
	PM (PI4P, PI(4,5)P_2_) [189]	Ca^2+^ [190]		PI4P→PI transfer (PM→ER) [190]
				Attenuates SOCE [190]
ORP5/8	ER [191,192]			Sterol, PS transfer (ER→PM) [144,191,193]
	PM (PI(4,5)P_2_) [194]			PI4P→PI transfer (PM→ER) [145,195]
ORP6	ER (VAP) [143]		VAP [143]	PI4P→PI transfer (PM→ER) [143]
PI4K	PM [196]			PI→PI4P [196]
PIP5K	PM [197]	Sterol/PS/PI4P nanodomains [150]		PI4P→PI(4,5)P_2_ [197]
		ORP [143,150]		
PIS	ER [198]			CDP-DAG→PI [198]
PKA	PM (AKAP) [172]	cAMP [199]	AKAP [101]	
PLC	PM [200]	Ca^2+^ [200]		PI(4,5)P2→DAG + IP_3_ [200]
Sac1	ER [148]			PI4P phosphatase [201]
SARAF	ER [83]		STIM1 [83]	Attenuates SOCE [83,202]
	PM [84]		Orai1 [88]	
			TRPC1 [86]	
Septin4	PM (PI(4,5)P_2_^)^ [203]			Promote STIM1—Orai1 assembly formation [154]
Septin7	PM (PI(4,5)P_2_) [203]			Prevents STIM1—Orai1 assembly formation [152,155]
SERCA	ER [204]	Ca^2+^ [204]		Inward-rectifying Ca^2+^ channel [204]
		Phospholamban (inhibitory) [205]		
STIM1	ER [47]	ER Ca^2+^ release [206,207]	Orai1 [184,185,186]	ER-localized Ca^2+^ sensor [206,207]
	PM (PI(4.5)P_2_) [208]	EB1 (inhibitory) [170,209] GRAMD2a [176]	TRPC [210]	
		SARAF (inhibitory) [83,88]	EB1 [168]	
TMEM24	ER [211]	Ca^2+^(inhibitory) [211,212]		PI transfer ER→PM [211]
	PM (PS) [211]	PLC (inhibitory) [212]		
TRPC1	PM [213,214]		STIM1 [215]	PM-localized non-selective cation channels
VAP	ER [187]		Nir2 [180]	Anchoring protein [216]
			ORPs [187]	

## References

[1] Raffaello A., Mammucari C., Gherardi G., Rizzuto R. (2016). Calcium at the Center of Cell Signaling: Interplay between Endoplasmic Reticulum, Mitochondria, and Lysosomes. Trends Biochem. Sci..

[2] Berridge M.J. (2016). The Inositol Trisphosphate/Calcium Signaling Pathway in Health and Disease. Physiol. Rev..

[3] Gold M.G., Gonen T., Scott J.D. (2013). Local CAMP Signaling in Disease at a Glance. J. Cell Sci..

[4] Ahuja M., Jha A., Maléth J., Park S., Muallem S. (2014). CAMP and Ca^2+^ Signaling in Secretory Epithelia: Crosstalk and Synergism. Cell Calcium.

[5] Maléth J., Hegyi P. (2016). Ca^2+^ Toxicity and Mitochondrial Damage in Acute Pancreatitis: Translational Overview. Philos. Trans. R. Soc. Lond. B Biol. Sci..

[6] Madácsy T., Pallagi P., Maleth J. (2018). Cystic Fibrosis of the Pancreas: The Role of CFTR Channel in the Regulation of Intracellular Ca^2+^ Signaling and Mitochondrial Function in the Exocrine Pancreas. Front. Physiol..

[7] Son A., Ahuja M., Schwartz D.M., Varga A., Swaim W., Kang N., Maleth J., Shin D.M., Muallem S. (2019). Ca^2+^ Influx Channel Inhibitor SARAF Protects Mice from Acute Pancreatitis. Gastroenterology.

[8] Pallagi P., Madácsy T., Varga Á., Maléth J. (2020). Intracellular Ca^2+^ Signalling in the Pathogenesis of Acute Pancreatitis: Recent Advances and Translational Perspectives. Int. J. Mol. Sci..

[9] Parton R.G., del Pozo M.A. (2013). Caveolae as Plasma Membrane Sensors, Protectors and Organizers. Nat. Rev. Mol. Cell Biol..

[10] Sezgin E., Levental I., Mayor S., Eggeling C. (2017). The Mystery of Membrane Organization: Composition, Regulation and Roles of Lipid Rafts. Nat. Rev. Mol. Cell Biol..

[11] Cao X., Choi S., Maléth J.J., Park S., Ahuja M., Muallem S. (2015). The ER/PM Microdomain, PI(4,5)P₂ and the Regulation of STIM1-Orai1 Channel Function. Cell Calcium.

[12] Prinz W.A., Toulmay A., Balla T. (2020). The Functional Universe of Membrane Contact Sites. Nat. Rev. Mol. Cell Biol..

[13] Valm A.M., Cohen S., Legant W.R., Melunis J., Hershberg U., Wait E., Cohen A.R., Davidson M.W., Betzig E., Lippincott-Schwartz J. (2017). Applying Systems-Level Spectral Imaging and Analysis to Reveal the Organelle Interactome. Nature.

[14] Shai N., Yifrach E., van Roermund C.W.T., Cohen N., Bibi C., IJlst L., Cavellini L., Meurisse J., Schuster R., Zada L. (2018). Systematic Mapping of Contact Sites Reveals Tethers and a Function for the Peroxisome-Mitochondria Contact. Nat. Commun..

[15] Schwarz D.S., Blower M.D. (2016). The Endoplasmic Reticulum: Structure, Function and Response to Cellular Signaling. Cell Mol. Life Sci..

[16] Parekh A.B., Putney J.W. (2005). Store-Operated Calcium Channels. Physiol. Rev..

[17] Weis W.I., Kobilka B.K. (2018). The Molecular Basis of G Protein-Coupled Receptor Activation. Annu. Rev. Biochem..

[18] Lopez J.J., Jardin I., Sanchez-Collado J., Salido G.M., Smani T., Rosado J.A. (2020). TRPC Channels in the SOCE Scenario. Cells.

[19] Choi S., Maleth J., Jha A., Lee K.P., Kim M.S., So I., Ahuja M., Muallem S. (2014). The TRPCs-STIM1-Orai Interaction. Handb. Exp. Pharmacol..

[20] Baba Y., Hayashi K., Fujii Y., Mizushima A., Watarai H., Wakamori M., Numaga T., Mori Y., Iino M., Hikida M. (2006). Coupling of STIM1 to Store-Operated Ca^2+^ Entry through Its Constitutive and Inducible Movement in the Endoplasmic Reticulum. Proc. Natl. Acad. Sci. USA.

[21] Derler I., Fahrner M., Muik M., Lackner B., Schindl R., Groschner K., Romanin C. (2009). A Ca^2+^ Release-Activated Ca^2+^ (CRAC) Modulatory Domain (CMD) within STIM1 Mediates Fast Ca^2+^-Dependent Inactivation of ORAI1 Channels. J. Biol. Chem..

[22] Jardin I., Dionisio N., Frischauf I., Berna-Erro A., Woodard G.E., López J.J., Salido G.M., Rosado J.A. (2013). The Polybasic Lysine-Rich Domain of Plasma Membrane-Resident STIM1 Is Essential for the Modulation of Store-Operated Divalent Cation Entry by Extracellular Calcium. Cell. Signal..

[23] Li Z., Lu J., Xu P., Xie X., Chen L., Xu T. (2007). Mapping the Interacting Domains of STIM1 and Orai1 in Ca^2+^ Release-Activated Ca^2+^ Channel Activation. J. Biol. Chem..

[24] Muik M., Frischauf I., Derler I., Fahrner M., Bergsmann J., Eder P., Schindl R., Hesch C., Polzinger B., Fritsch R. (2008). Dynamic Coupling of the Putative Coiled-Coil Domain of ORAI1 with STIM1 Mediates ORAI1 Channel Activation. J. Biol. Chem..

[25] Stathopulos P.B., Zheng L., Li G.-Y., Plevin M.J., Ikura M. (2008). Structural and Mechanistic Insights into STIM1-Mediated Initiation of Store-Operated Calcium Entry. Cell.

[26] Yuan J.P., Zeng W., Dorwart M.R., Choi Y.-J., Worley P.F., Muallem S. (2009). SOAR and the Polybasic STIM1 Domains Gate and Regulate Orai Channels. Nat. Cell Biol..

[27] Zheng L., Stathopulos P.B., Li G.-Y., Ikura M. (2008). Biophysical Characterization of the EF-Hand and SAM Domain Containing Ca^2+^ Sensory Region of STIM1 and STIM2. Biochem. Biophys. Res. Commun..

[28] Stathopulos P.B., Li G.-Y., Plevin M.J., Ames J.B., Ikura M. (2006). Stored Ca^2+^ Depletion-Induced Oligomerization of Stromal Interaction Molecule 1 (STIM1) via the EF-SAM Region. J. Biol. Chem..

[29] Ma G., Wei M., He L., Liu C., Wu B., Zhang S.L., Jing J., Liang X., Senes A., Tan P. (2015). Inside-out Ca^2+^ Signalling Prompted by STIM1 Conformational Switch. Nat. Commun..

[30] Dong H., Fiorin G., Carnevale V., Treptow W., Klein M.L. (2013). Pore Waters Regulate Ion Permeation in a Calcium Release-Activated Calcium Channel. Proc. Natl. Acad. Sci. USA.

[31] Hirve N., Rajanikanth V., Hogan P.G., Gudlur A. (2018). Coiled-Coil Formation Conveys a STIM1 Signal from ER Lumen to Cytoplasm. Cell Rep..

[32] Huang G.N., Zeng W., Kim J.Y., Yuan J.P., Han L., Muallem S., Worley P.F. (2006). STIM1 Carboxyl-Terminus Activates Native SOC, Icrac and TRPC1 Channels. Nat. Cell Biol..

[33] Muik M., Fahrner M., Derler I., Schindl R., Bergsmann J., Frischauf I., Groschner K., Romanin C. (2009). A Cytosolic Homomerization and a Modulatory Domain within STIM1 C Terminus Determine Coupling to ORAI1 Channels. J. Biol. Chem..

[34] Yang X., Jin H., Cai X., Li S., Shen Y. (2012). Structural and Mechanistic Insights into the Activation of Stromal Interaction Molecule 1 (STIM1). Proc. Natl. Acad. Sci. USA.

[35] Park C.Y., Hoover P.J., Mullins F.M., Bachhawat P., Covington E.D., Raunser S., Walz T., Garcia K.C., Dolmetsch R.E., Lewis R.S. (2009). STIM1 Clusters and Activates CRAC Channels via Direct Binding of a Cytosolic Domain to Orai1. Cell.

[36] Muik M., Fahrner M., Schindl R., Stathopulos P., Frischauf I., Derler I., Plenk P., Lackner B., Groschner K., Ikura M. (2011). STIM1 Couples to ORAI1 via an Intramolecular Transition into an Extended Conformation. EMBO J..

[37] Kawasaki T., Lange I., Feske S. (2009). A Minimal Regulatory Domain in the C Terminus of STIM1 Binds to and Activates ORAI1 CRAC Channels. Biochem. Biophys. Res. Commun..

[38] Soboloff J., Rothberg B.S., Madesh M., Gill D.L. (2012). STIM Proteins: Dynamic Calcium Signal Transducers. Nat. Rev. Mol. Cell Biol..

[39] Fahrner M., Muik M., Schindl R., Butorac C., Stathopulos P., Zheng L., Jardin I., Ikura M., Romanin C. (2014). A Coiled-Coil Clamp Controls Both Conformation and Clustering of Stromal Interaction Molecule 1 (STIM1). J. Biol. Chem..

[40] Zhou Y., Srinivasan P., Razavi S., Seymour S., Meraner P., Gudlur A., Stathopulos P.B., Ikura M., Rao A., Hogan P.G. (2013). Initial Activation of STIM1, the Regulator of Store-Operated Calcium Entry. Nat. Struct. Mol. Biol..

[41] Liou J., Fivaz M., Inoue T., Meyer T. (2007). Live-Cell Imaging Reveals Sequential Oligomerization and Local Plasma Membrane Targeting of Stromal Interaction Molecule 1 after Ca^2+^ Store Depletion. Proc. Natl. Acad. Sci. USA.

[42] Zheng S., Zhou L., Ma G., Zhang T., Liu J., Li J., Nguyen N.T., Zhang X., Li W., Nwokonko R. (2018). Calcium Store Refilling and STIM Activation in STIM- and Orai-Deficient Cell Lines. Pflug. Arch..

[43] Wu M.M., Covington E.D., Lewis R.S. (2014). Single-Molecule Analysis of Diffusion and Trapping of STIM1 and Orai1 at Endoplasmic Reticulum–Plasma Membrane Junctions. Mol. Biol. Cell.

[44] Williams R.T., Manji S.S.M., Parker N.J., Hancock M.S., van Stekelenburg L., Eid J.-P., Senior P.V., Kazenwadel J.S., Shandala T., Saint R. (2001). Identification and Characterization of the STIM (Stromal Interaction Molecule) Gene Family: Coding for a Novel Class of Transmembrane Proteins. Biochem. J..

[45] Lopez E., Jardin I., Berna-Erro A., Bermejo N., Salido G.M., Sage S.O., Rosado J.A., Redondo P.C. (2012). STIM1 Tyrosine-Phosphorylation Is Required for STIM1-Orai1 Association in Human Platelets. Cell. Signal..

[46] Lopez E., Frischauf I., Jardin I., Derler I., Muik M., Cantonero C., Salido G.M., Smani T., Rosado J.A., Redondo P.C. (2019). STIM1 Phosphorylation at Y316 Modulates Its Interaction with SARAF and the Activation of SOCE and ICRAC. J. Cell Sci..

[47] Williams R.T., Senior P.V., Van Stekelenburg L., Layton J.E., Smith P.J., Dziadek M.A. (2002). Stromal Interaction Molecule 1 (STIM1), a Transmembrane Protein with Growth Suppressor Activity, Contains an Extracellular SAM Domain Modified by N-Linked Glycosylation. Biochim. Et Biophys. Acta (BBA)-Protein Struct. Mol. Enzymol..

[48] Darbellay B., Arnaudeau S., Bader C.R., Konig S., Bernheim L. (2011). STIM1L Is a New Actin-Binding Splice Variant Involved in Fast Repetitive Ca^2+^ Release. J. Cell Biol..

[49] Horinouchi T., Higashi T., Higa T., Terada K., Mai Y., Aoyagi H., Hatate C., Nepal P., Horiguchi M., Harada T. (2012). Different Binding Property of STIM1 and Its Novel Splice Variant STIM1L to Orai1, TRPC3, and TRPC6 Channels. Biochem. Biophys. Res. Commun..

[50] Rana A., Yen M., Sadaghiani A.M., Malmersjö S., Park C.Y., Dolmetsch R.E., Lewis R.S. (2015). Alternative Splicing Converts STIM2 from an Activator to an Inhibitor of Store-Operated Calcium Channels. J. Cell Biol..

[51] Miederer A.-M., Alansary D., Schwär G., Lee P.-H., Jung M., Helms V., Niemeyer B.A. (2015). A STIM2 Splice Variant Negatively Regulates Store-Operated Calcium Entry. Nat. Commun..

[52] Brandman O., Liou J., Park W.S., Meyer T. (2007). STIM2 Is a Feedback Regulator That Stabilizes Basal Cytosolic and Endoplasmic Reticulum Ca^2+^ Levels. Cell.

[53] Zhou Y., Mancarella S., Wang Y., Yue C., Ritchie M., Gill D.L., Soboloff J. (2009). The Short N-Terminal Domains of STIM1 and STIM2 Control the Activation Kinetics of Orai1 Channels. J. Biol. Chem..

[54] Berna-Erro A., Jardin I., Salido G.M., Rosado J.A. (2017). Role of STIM2 in Cell Function and Physiopathology. J. Physiol..

[55] Cai X., Zhou Y., Nwokonko R.M., Loktionova N.A., Wang X., Xin P., Trebak M., Wang Y., Gill D.L. (2016). The Orai1 Store-Operated Calcium Channel Functions as a Hexamer. J. Biol. Chem..

[56] Hou X., Pedi L., Diver M.M., Long S.B. (2012). Crystal Structure of the Calcium Release-Activated Calcium Channel Orai. Science.

[57] Rothberg B.S., Wang Y., Gill D.L. (2013). Orai Channel Pore Properties and Gating by STIM: Implications from the Orai Crystal Structure. Sci. Signal..

[58] Yen M., Lokteva L.A., Lewis R.S. (2016). Functional Analysis of Orai1 Concatemers Supports a Hexameric Stoichiometry for the CRAC Channel. Biophys. J..

[59] Zhou Y., Cai X., Loktionova N.A., Wang X., Nwokonko R.M., Wang X., Wang Y., Rothberg B.S., Trebak M., Gill D.L. (2016). The STIM1-Binding Site Nexus Remotely Controls Orai1 Channel Gating. Nat. Commun..

[60] Frischauf I., Muik M., Derler I., Bergsmann J., Fahrner M., Schindl R., Groschner K., Romanin C. (2009). Molecular Determinants of the Coupling between STIM1 and Orai Channels: Differential activation of Orai1–3 Channels by a STIM1 coiled-coil mutant. J. Biol. Chem..

[61] Frischauf I., Schindl R., Bergsmann J., Derler I., Fahrner M., Muik M., Fritsch R., Lackner B., Groschner K., Romanin C. (2011). Cooperativeness of Orai Cytosolic Domains Tunes Subtype-Specific Gating. J. Biol. Chem..

[62] Gudlur A., Quintana A., Zhou Y., Hirve N., Mahapatra S., Hogan P.G. (2014). STIM1 Triggers a Gating Rearrangement at the Extracellular Mouth of the ORAI1 Channel. Nat. Commun..

[63] Niu L., Wu F., Li K., Li J., Zhang S.L., Hu J., Wang Q. (2020). STIM1 Interacts with Termini of Orai Channels in a Sequential Manner. J. Cell Sci..

[64] Peckys D.B., Gaa D., Alansary D., Niemeyer B.A., de Jonge N. (2021). Supra-Molecular Assemblies of ORAI1 at Rest Precede Local Accumulation into Puncta after Activation. Int. J. Mol. Sci..

[65] McNally B.A., Somasundaram A., Jairaman A., Yamashita M., Prakriya M. (2013). The C- and N-Terminal STIM1 Binding Sites on Orai1 Are Required for Both Trapping and Gating CRAC Channels. J. Physiol..

[66] Palty R., Stanley C., Isacoff E.Y. (2015). Critical Role for Orai1 C-Terminal Domain and TM4 in CRAC Channel Gating. Cell Res..

[67] Zheng H., Zhou M.-H., Hu C., Kuo E., Peng X., Hu J., Kuo L., Zhang S.L. (2013). Differential Roles of the C and N Termini of Orai1 Protein in Interacting with Stromal Interaction Molecule 1 (STIM1) for Ca^2+^ Release-Activated Ca^2+^ (CRAC) Channel Activation*. J. Biol. Chem..

[68] Baraniak J.H., Zhou Y., Nwokonko R.M., Gill D.L. (2020). The Intricate Coupling between STIM Proteins and Orai Channels. Curr. Opin. Physiol..

[69] Hou X., Outhwaite I.R., Pedi L., Long S.B. (2020). Cryo-EM Structure of the Calcium Release-Activated Calcium Channel Orai in an Open Conformation. eLife.

[70] Liu X., Wu G., Yu Y., Chen X., Ji R., Lu J., Li X., Zhang X., Yang X., Shen Y. (2019). Molecular Understanding of Calcium Permeation through the Open Orai Channel. PLoS Biol..

[71] Tiffner A., Schober R., Hoeglinger C., Bonhenry D., Pandey S., Lunz V., Sallinger M., Frischauf I., Fahrner M., Lindinger S. (2020). CRAC Channel Opening Is Determined by a Series of Orai1 Gating Checkpoints in the Transmembrane and Cytosolic Regions. J. Biol. Chem..

[72] Pedersen S.F., Owsianik G., Nilius B. (2005). TRP Channels: An Overview. Cell Calcium.

[73] Freichel M., Vennekens R., Olausson J., Stolz S., Philipp S.E., Weißgerber P., Flockerzi V. (2005). Functional Role of TRPC Proteins in Native Systems: Implications from Knockout and Knock-down Studies. J. Physiol..

[74] Yuan J., Zeng W., Huang G., Worley P., Muallem S. (2007). STIM1 Heteromultimerizes TRPC Channels to Determine Their Function as Store-Operated Channels. Nat. Cell Biol..

[75] Liao Y., Erxleben C., Abramowitz J., Flockerzi V., Zhu M.X., Armstrong D.L., Birnbaumer L. (2008). Functional Interactions among Orai1, TRPCs, and STIM1 Suggest a STIM-Regulated Heteromeric Orai/TRPC Model for SOCE/Icrac Channels. Proc. Natl. Acad. Sci. USA.

[76] Liao Y., Erxleben C., Yildirim E., Abramowitz J., Armstrong D.L., Birnbaumer L. (2007). Orai Proteins Interact with TRPC Channels and Confer Responsiveness to Store Depletion. Proc. Natl. Acad. Sci. USA.

[77] Liao Y., Plummer N.W., George M.D., Abramowitz J., Zhu M.X., Birnbaumer L. (2009). A Role for Orai in TRPC-Mediated Ca^2+^ Entry Suggests That a TRPC:Orai Complex May Mediate Store and Receptor Operated Ca^2+^ Entry. Proc. Natl. Acad. Sci. USA.

[78] Kim M.S., Zeng W., Yuan J.P., Shin D.M., Worley P.F., Muallem S. (2009). Native Store-Operated Ca^2+^ Influx Requires the Channel Function of Orai1 and TRPC1. J. Biol. Chem..

[79] Cheng K.T., Liu X., Ong H.L., Swaim W., Ambudkar I.S. (2011). Local Ca^2+^ Entry via Orai1 Regulates Plasma Membrane Recruitment of TRPC1 and Controls Cytosolic Ca^2+^ Signals Required for Specific Cell Functions. PLoS Biol..

[80] Ong H.L., Cheng K.T., Liu X., Bandyopadhyay B.C., Paria B.C., Soboloff J., Pani B., Gwack Y., Srikanth S., Singh B.B. (2007). Dynamic Assembly of TRPC1-STIM1-Orai1 Ternary Complex Is Involved in Store-Operated Calcium Influx. J. Biol. Chem..

[81] Zeng W., Yuan J.P., Kim M.S., Choi Y.J., Huang G.N., Worley P.F., Muallem S. (2008). STIM1 Gates TRPC Channels, but Not Orai1, by Electrostatic Interaction. Mol. Cell.

[82] Albarran L., Lopez J.J., Jardin I., Sanchez-Collado J., Berna-Erro A., Smani T., Camello P.J., Salido G.M., Rosado J.A. (2018). EFHB Is a Novel Cytosolic Ca^2+^ Sensor That Modulates STIM1-SARAF Interaction. Cell Physiol. Biochem..

[83] Palty R., Raveh A., Kaminsky I., Meller R., Reuveny E. (2012). SARAF Inactivates the Store Operated Calcium Entry Machinery to Prevent Excess Calcium Refilling. Cell.

[84] Albarran L., Regodón S., Salido G.M., Lopez J.J., Rosado J.A. (2016). Role of STIM1 in the Surface Expression of SARAF. Channels.

[85] Jha A., Ahuja M., Maléth J., Moreno C.M., Yuan J.P., Kim M.S., Muallem S. (2013). The STIM1 CTID Domain Determines Access of SARAF to SOAR to Regulate Orai1 Channel Function. J. Cell Biol..

[86] Albarrán L., López J.J., Gómez L.J., Salido G.M., Rosado J.A. (2016). SARAF Modulates TRPC1, but Not TRPC6, Channel Function in a STIM1-Independent Manner. Biochem. J..

[87] Li X., Wu G., Yang Y., Fu S., Liu X., Kang H., Yang X., Su X.-C., Shen Y. (2017). Calmodulin Dissociates the STIM1-Orai1 Complex and STIM1 Oligomers. Nat. Commun..

[88] Albarran L., Lopez J.J., Amor N.B., Martin-Cano F.E., Berna-Erro A., Smani T., Salido G.M., Rosado J.A. (2016). Dynamic Interaction of SARAF with STIM1 and Orai1 to Modulate Store-Operated Calcium Entry. Sci. Rep..

[89] Bruce J.I.E., Straub S.V., Yule D.I. (2003). Crosstalk between CAMP and Ca^2+^ Signaling in Non-Excitable Cells. Cell Calcium.

[90] Lee K.P., Yuan J.P., Hong J.H., So I., Worley P.F., Muallem S. (2010). An Endoplasmic Reticulum/Plasma Membrane Junction: STIM1/Orai1/TRPCs. FEBS Lett..

[91] Zhang B.X., Zhao H., Loessberg P., Muallem S. (1992). Activation of the Plasma Membrane Ca^2+^ Pump during Agonist Stimulation of Pancreatic Acini. J. Biol. Chem..

[92] Boyajian C.L., Garritsen A., Cooper D.M. (1991). Bradykinin Stimulates Ca^2+^ Mobilization in NCB-20 Cells Leading to Direct Inhibition of Adenylylcyclase. A Novel Mechanism for Inhibition of CAMP Production. J. Biol. Chem..

[93] Fagan K.A., Mahey R., Cooper D.M. (1996). Functional Co-Localization of Transfected Ca(2+)-Stimulable Adenylyl Cyclases with Capacitative Ca^2+^ Entry Sites. J. Biol. Chem..

[94] Halls M.L., Cooper D.M.F. (2011). Regulation by Ca^2+^-Signaling Pathways of Adenylyl Cyclases. Cold Spring Harb. Perspect. Biol..

[95] Masada N., Ciruela A., MacDougall D.A., Cooper D.M.F. (2009). Distinct Mechanisms of Regulation by Ca^2+^/Calmodulin of Type 1 and 8 Adenylyl Cyclases Support Their Different Physiological Roles. J. Biol. Chem..

[96] Shuttleworth T.J., Thompson J.L. (1999). Discriminating between Capacitative and Arachidonate-Activated Ca(2+) Entry Pathways in HEK293 Cells. J. Biol. Chem..

[97] Watson E.L., Wu Z., Jacobson K.L., Storm D.R., Singh J.C., Ott S.M. (1998). Capacitative Ca^2+^ Entry Is Involved in CAMP Synthesis in Mouse Parotid Acini. Am. J. Physiol. Cell Physiol..

[98] Martin A.C.L., Cooper D.M.F. (2006). Capacitative and 1-Oleyl-2-Acetyl-Sn-Glycerol-Activated Ca(2+) Entry Distinguished Using Adenylyl Cyclase Type 8. Mol. Pharm..

[99] Willoughby D., Everett K.L., Halls M.L., Pacheco J., Skroblin P., Vaca L., Klussmann E., Cooper D.M.F. (2012). Direct Binding Between Orai1 and AC8 Mediates Dynamic Interplay Between Ca^2+^ and CAMP Signaling. Sci. Signal..

[100] Willoughby D., Ong H.L., De Souza L.B., Wachten S., Ambudkar I.S., Cooper D.M.F. (2014). TRPC1 Contributes to the Ca^2+^ -Dependent Regulation of Adenylate Cyclases. Biochem. J..

[101] Michel J.J.C., Scott J.D. (2002). AKAP Mediated Signal Transduction. Annu. Rev. Pharmacol. Toxicol..

[102] Torres-Quesada O., Mayrhofer J.E., Stefan E. (2017). The Many Faces of Compartmentalized PKA Signalosomes. Cell. Signal..

[103] Johnstone T.B., Agarwal S.R., Harvey R.D., Ostrom R.S. (2018). CAMP Signaling Compartmentation: Adenylyl Cyclases as Anchors of Dynamic Signaling Complexes. Mol. Pharm..

[104] Müller M.R., Rao A. (2010). NFAT, Immunity and Cancer: A Transcription Factor Comes of Age. Nat. Rev. Immunol..

[105] Wu H., Peisley A., Graef I.A., Crabtree G.R. (2007). NFAT Signaling and the Invention of Vertebrates. Trends Cell Biol..

[106] Kar P., Samanta K., Kramer H., Morris O., Bakowski D., Parekh A.B. (2014). Dynamic Assembly of a Membrane Signaling Complex Enables Selective Activation of NFAT by Orai1. Curr. Biol..

[107] Son G.-Y., Subedi K.P., Ong H.L., Noyer L., Saadi H., Zheng C., Bhardwaj R., Feske S., Ambudkar I.S. (2020). STIM2 Targets Orai1/STIM1 to the AKAP79 Signaling Complex and Confers Coupling of Ca^2+^ Entry with NFAT1 Activation. Proc. Natl. Acad. Sci. USA.

[108] Wei J., Zhao A.Z., Chan G.C., Baker L.P., Impey S., Beavo J.A., Storm D.R. (1998). Phosphorylation and Inhibition of Olfactory Adenylyl Cyclase by CaM Kinase II in Neurons: A Mechanism for Attenuation of Olfactory Signals. Neuron.

[109] Motiani R.K., Tanwar J., Raja D.A., Vashisht A., Khanna S., Sharma S., Srivastava S., Sivasubbu S., Natarajan V.T., Gokhale R.S. (2018). STIM1 Activation of Adenylyl Cyclase 6 Connects Ca^2+^ and CAMP Signaling during Melanogenesis. EMBO J..

[110] Maus M., Cuk M., Patel B., Lian J., Ouimet M., Kaufmann U., Yang J., Horvath R., Hornig-Do H.-T., Chrzanowska-Lightowlers Z. (2017). Store-Operated Ca^2+^ Entry (SOCE) Controls Induction of Lipolysis and the Transcriptional Reprogramming to Lipid Metabolism. Cell Metab..

[111] Lefkimmiatis K., Srikanthan M., Maiellaro I., Moyer M.P., Curci S., Hofer A.M. (2009). Store-Operated Cyclic AMP Signalling Mediated by STIM1. Nat. Cell Biol..

[112] Hofer A.M. (2012). Interactions between Calcium and CAMP Signaling. Curr. Med. Chem..

[113] Spirli C., Locatelli L., Fiorotto R., Morell C.M., Fabris L., Pozzan T., Strazzabosco M. (2012). Altered Store Operated Calcium Entry Increases Cyclic 3’,5’-Adenosine Monophosphate Production and Extracellular Signal-Regulated Kinases 1 and 2 Phosphorylation in Polycystin-2-Defective Cholangiocytes. Hepatology.

[114] Tian G., Tepikin A.V., Tengholm A., Gylfe E. (2012). CAMP Induces Stromal Interaction Molecule 1 (STIM1) Puncta but Neither Orai1 Protein Clustering nor Store-Operated Ca^2+^ Entry (SOCE) in Islet Cells. J. Biol. Chem..

[115] Garcia-Alvarez G., Lu B., Yap K.A.F., Wong L.C., Thevathasan J.V., Lim L., Ji F., Tan K.W., Mancuso J.J., Tang W. (2015). STIM2 Regulates PKA-Dependent Phosphorylation and Trafficking of AMPARs. Mol. Biol. Cell.

[116] Bender A.T., Beavo J.A. (2006). Cyclic Nucleotide Phosphodiesterases: Molecular Regulation to Clinical Use. Pharm. Rev..

[117] Jeon K.-I., Jono H., Miller C.L., Cai Y., Lim S., Liu X., Gao P., Abe J.-I., Li J.-D., Yan C. (2010). Ca^2+^/Calmodulin-Stimulated PDE1 Regulates the Beta-Catenin/TCF Signaling through PP2A B56 Gamma Subunit in Proliferating Vascular Smooth Muscle Cells. FEBS J..

[118] Kincaid R.L., Stith-Coleman I.E., Vaughan M. (1985). Proteolytic Activation of Calmodulin-Dependent Cyclic Nucleotide Phosphodiesterase. J. Biol. Chem..

[119] Sonnenburg W.K., Seger D., Kwak K.S., Huang J., Charbonneau H., Beavo J.A. (1995). Identification of Inhibitory and Calmodulin-Binding Domains of the PDE1A1 and PDE1A2 Calmodulin-Stimulated Cyclic Nucleotide Phosphodiesterases. J. Biol. Chem..

[120] Goraya T.A., Masada N., Ciruela A., Willoughby D., Clynes M.A., Cooper D.M.F. (2008). Kinetic Properties of Ca^2+^/Calmodulin-Dependent Phosphodiesterase Isoforms Dictate Intracellular CAMP Dynamics in Response to Elevation of Cytosolic Ca^2+^. Cell. Signal..

[121] Wagner L.E., Joseph S.K., Yule D.I. (2008). Regulation of Single Inositol 1,4,5-Trisphosphate Receptor Channel Activity by Protein Kinase A Phosphorylation. J. Physiol..

[122] Wagner L.E., Li W.-H., Yule D.I. (2003). Phosphorylation of Type-1 Inositol 1,4,5-Trisphosphate Receptors by Cyclic Nucleotide-Dependent Protein Kinases: A Mutational Analysis of the Functionally Important Sites in the S2+ and S2- Splice Variants. J. Biol. Chem..

[123] Wagner L.E., Li W.-H., Joseph S.K., Yule D.I. (2004). Functional Consequences of Phosphomimetic Mutations at Key CAMP-Dependent Protein Kinase Phosphorylation Sites in the Type 1 Inositol 1,4,5-Trisphosphate Receptor. J. Biol. Chem..

[124] Betzenhauser M.J., Fike J.L., Wagner L.E., Yule D.I. (2009). Protein Kinase a Increases Type-2 Inositol 1,4,5-Trisphosphate Receptor Activity by Phosphorylation of Serine 937. J. Biol. Chem..

[125] Park S., Shcheynikov N., Hong J.H., Zheng C., Suh S.H., Kawaai K., Ando H., Mizutani A., Abe T., Kiyonari H. (2013). Irbit Mediates Synergy Between Ca^2+^ and CAMP Signaling Pathways During Epithelial Transport in Mice. Gastroenterology.

[126] Zhang X., Pathak T., Yoast R., Emrich S., Xin P., Nwokonko R.M., Johnson M., Wu S., Delierneux C., Gueguinou M. (2019). A Calcium/CAMP Signaling Loop at the ORAI1 Mouth Drives Channel Inactivation to Shape NFAT Induction. Nat. Commun..

[127] Thompson J.L., Shuttleworth T.J. (2015). Anchoring Protein AKAP79-Mediated PKA Phosphorylation of STIM1 Determines Selective Activation of the ARC Channel, a Store-Independent Orai Channel. J. Physiol..

[128] Thompson J.L., Zhao Y., Stathopulos P.B., Grossfield A., Shuttleworth T.J. (2018). Phosphorylation-Mediated Structural Changes within the SOAR Domain of Stromal Interaction Molecule 1 Enable Specific Activation of Distinct Orai Channels. J. Biol. Chem..

[129] Cerra M.C., Imbrogno S. (2012). Phospholamban and Cardiac Function: A Comparative Perspective in Vertebrates. Acta Physiol..

[130] Bruce J.I.E., Yule D.I., Shuttleworth T.J. (2002). Ca^2+^-Dependent Protein Kinase--a Modulation of the Plasma Membrane Ca^2+^-ATPase in Parotid Acinar Cells. J. Biol. Chem..

[131] Stefan C.J. (2020). Endoplasmic Reticulum–Plasma Membrane Contacts: Principals of Phosphoinositide and Calcium Signaling. Curr. Opin. Cell Biol..

[132] Vance J.E. (2020). Inter-Organelle Membrane Contact Sites: Implications for Lipid Metabolism. Biol. Direct.

[133] Scorrano L., De Matteis M.A., Emr S., Giordano F., Hajnóczky G., Kornmann B., Lackner L.L., Levine T.P., Pellegrini L., Reinisch K. (2019). Coming Together to Define Membrane Contact Sites. Nat. Commun..

[134] Toulmay A., Prinz W.A. (2012). A Conserved Membrane-Binding Domain Targets Proteins to Organelle Contact Sites. J. Cell Sci..

[135] Min S.-W., Chang W.-P., Südhof T.C. (2007). E-Syts, a Family of Membranous Ca^2+^-Sensor Proteins with Multiple C2 Domains. Proc. Natl. Acad. Sci. USA.

[136] Giordano F., Saheki Y., Idevall-Hagren O., Colombo S.F., Pirruccello M., Milosevic I., Gracheva E.O., Bagriantsev S.N., Borgese N., De Camilli P. (2013). PI(4,5)P2-Dependent and Ca^2+^-Regulated ER-PM Interactions Mediated by the Extended Synaptotagmins. Cell.

[137] Schauder C.M., Wu X., Saheki Y., Narayanaswamy P., Torta F., Wenk M.R., De Camilli P., Reinisch K.M. (2014). Structure of a Lipid-Bound Extended-Synaptotagmin Indicates a Role in Lipid Transfer. Nature.

[138] Chang C.-L., Hsieh T.-S., Yang T.T., Rothberg K.G., Azizoglu D.B., Volk E., Liao J.-C., Liou J. (2013). Feedback Regulation of Receptor-Induced Ca^2+^ Signaling Mediated by E-Syt1 and Nir2 at Endoplasmic Reticulum-Plasma Membrane Junctions. Cell Rep..

[139] Idevall-Hagren O., Lü A., Xie B., De Camilli P. (2015). Triggered Ca^2+^ Influx Is Required for Extended Synaptotagmin 1-Induced ER-Plasma Membrane Tethering. EMBO J..

[140] Fernández-Busnadiego R., Saheki Y., De Camilli P. (2015). Three-Dimensional Architecture of Extended Synaptotagmin-Mediated Endoplasmic Reticulum–Plasma Membrane Contact Sites. Proc. Natl. Acad. Sci. USA.

[141] Saheki Y., Bian X., Schauder C.M., Sawaki Y., Surma M.A., Klose C., Pincet F., Reinisch K.M., De Camilli P. (2016). Control of Plasma Membrane Lipid Homeostasis by the Extended Synaptotagmins. Nat. Cell Biol..

[142] Hsieh T.-S., Chen Y.-J., Chang C.-L., Lee W.-R., Liou J. (2017). Cortical Actin Contributes to Spatial Organization of ER–PM Junctions. Mol. Biol. Cell.

[143] Mochizuki S., Miki H., Zhou R., Kido Y., Nishimura W., Kikuchi M., Noda Y. (2018). Oxysterol-Binding Protein-Related Protein (ORP) 6 Localizes to the ER and ER-Plasma Membrane Contact Sites and Is Involved in the Turnover of PI4P in Cerebellar Granule Neurons. Exp. Cell Res..

[144] Chung J., Torta F., Masai K., Lucast L., Czapla H., Tanner L.B., Narayanaswamy P., Wenk M.R., Nakatsu F., De Camilli P. (2015). Intracellular transport. PI4P/Phosphatidylserine Countertransport at ORP5- and ORP8-Mediated ER-Plasma Membrane contacts. Science.

[145] Moser von Filseck J., Čopič A., Delfosse V., Vanni S., Jackson C.L., Bourguet W., Drin G. (2015). Intracellular transport. Phosphatidylserine Transport by ORP/Osh Proteins Is Driven by Phosphatidylinositol 4-Phosphate. Science.

[146] Moser von Filseck J.M., Vanni S., Mesmin B., Antonny B., Drin G. (2015). A Phosphatidylinositol-4-Phosphate Powered Exchange Mechanism to Create a Lipid Gradient between Membranes. Nat. Commun..

[147] Schulz T.A., Choi M.-G., Raychaudhuri S., Mears J.A., Ghirlando R., Hinshaw J.E., Prinz W.A. (2009). Lipid-Regulated Sterol Transfer between Closely Apposed Membranes by Oxysterol-Binding Protein Homologues. J. Cell Biol..

[148] Tahirovic S., Schorr M., Mayinger P. (2005). Regulation of Intracellular Phosphatidylinositol-4-Phosphate by the Sac1 Lipid Phosphatase. Traffic.

[149] Naito T., Ercan B., Krshnan L., Triebl A., Koh D.H.Z., Wei F.-Y., Tomizawa K., Torta F.T., Wenk M.R., Saheki Y. (2019). Movement of Accessible Plasma Membrane Cholesterol by the GRAMD1 Lipid Transfer Protein Complex. eLife.

[150] Nishimura T., Gecht M., Covino R., Hummer G., Surma M.A., Klose C., Arai H., Kono N., Stefan C.J. (2019). Osh Proteins Control Nanoscale Lipid Organization Necessary for PI(4,5)P2 Synthesis. Mol. Cell.

[151] Sharma S., Quintana A., Findlay G.M., Mettlen M., Baust B., Jain M., Nilsson R., Rao A., Hogan P.G. (2013). An SiRNA Screen for NFAT Activation Identifies Septins as Coordinators of Store-Operated Ca^2+^ Entry. Nature.

[152] Deb B.K., Hasan G. (2019). SEPT7-Mediated Regulation of Ca^2+^ Entry through Orai Channels Requires Other Septin Subunits. Cytoskeleton.

[153] Deb B.K., Pathak T., Hasan G. (2016). Store-Independent Modulation of Ca^2+^ Entry through Orai by Septin 7. Nat. Commun..

[154] Katz Z.B., Zhang C., Quintana A., Lillemeier B.F., Hogan P.G. (2019). Septins Organize Endoplasmic Reticulum-Plasma Membrane Junctions for STIM1-ORAI1 Calcium Signalling. Sci. Rep..

[155] Deb B.K., Chakraborty P., Gopurappilly R., Hasan G. (2020). SEPT7 Regulates Ca^2+^ Entry through Orai Channels in Human Neural Progenitor Cells and Neurons. Cell Calcium.

[156] Maass K., Fischer M.A., Seiler M., Temmerman K., Nickel W., Seedorf M. (2009). A Signal Comprising a Basic Cluster and an Amphipathic α-Helix Interacts with Lipids and Is Required for the Transport of Ist2 to the Yeast Cortical ER. J. Cell Sci..

[157] Whitlock J.M., Hartzell H.C. (2017). Anoctamins/TMEM16 Proteins: Chloride Channels Flirting with Lipids and Extracellular Vesicles. Annu. Rev. Physiol..

[158] Dang S., Feng S., Tien J., Peters C.J., Bulkley D., Lolicato M., Zhao J., Zuberbühler K., Ye W., Qi L. (2017). Cryo-EM Structures of the TMEM16A Calcium-Activated Chloride Channel. Nature.

[159] Paulino C., Kalienkova V., Lam A.K.M., Neldner Y., Dutzler R. (2017). Activation Mechanism of the Calcium-Activated Chloride Channel TMEM16A Revealed by Cryo-EM. Nature.

[160] Huang W.C., Xiao S., Huang F., Harfe B.D., Jan Y.N., Jan L.Y. (2012). Calcium-Activated Chloride Channels (CaCCs) Regulate Action Potential and Synaptic Response in Hippocampal Neurons. Neuron.

[161] Stöhr H., Heisig J.B., Benz P.M., Schöberl S., Milenkovic V.M., Strauss O., Aartsen W.M., Wijnholds J., Weber B.H.F., Schulz H.L. (2009). TMEM16B, a Novel Protein with Calcium-Dependent Chloride Channel Activity, Associates with a Presynaptic Protein Complex in Photoreceptor Terminals. J. Neurosci..

[162] Suzuki J., Umeda M., Sims P.J., Nagata S. (2010). Calcium-Dependent Phospholipid Scrambling by TMEM16F. Nature.

[163] Brunner J.D., Lim N.K., Schenck S., Duerst A., Dutzler R. (2014). X-Ray Structure of a Calcium-Activated TMEM16 Lipid Scramblase. Nature.

[164] Alvadia C., Lim N.K., Clerico Mosina V., Oostergetel G.T., Dutzler R., Paulino C. (2019). Cryo-EM Structures and Functional Characterization of the Murine Lipid Scramblase TMEM16F. eLife.

[165] Jha A., Chung W.Y., Vachel L., Maleth J., Lake S., Zhang G., Ahuja M., Muallem S. (2019). Anoctamin 8 Tethers Endoplasmic Reticulum and Plasma Membrane for Assembly of Ca^2+^ Signaling Complexes at the ER/PM Compartment. EMBO J..

[166] Akhmanova A., Steinmetz M.O. (2010). Microtubule +TIPs at a Glance. J. Cell Sci..

[167] Asanov A., Sherry R., Sampieri A., Vaca L. (2013). A Relay Mechanism between EB1 and APC Facilitate STIM1 Puncta Assembly at Endoplasmic Reticulum–Plasma Membrane Junctions. Cell Calcium.

[168] Grigoriev I., Gouveia S.M., van der Vaart B., Demmers J., Smyth J.T., Honnappa S., Splinter D., Steinmetz M.O., Putney J.W., Hoogenraad C.C. (2008). STIM1 Is a Microtubule plus End Tracking Protein Involved in Remodeling of the Endoplasmic Reticulum. Curr. Biol..

[169] Honnappa S., Gouveia S.M., Weisbrich A., Damberger F.F., Bhavesh N.S., Jawhari H., Grigoriev I., van Rijssel F.J.A., Buey R.M., Lawera A. (2009). An EB1-Binding Motif Acts as a Microtubule Tip Localization Signal. Cell.

[170] Chang C.-L., Chen Y.-J., Quintanilla C.G., Hsieh T.-S., Liou J. (2018). EB1 Binding Restricts STIM1 Translocation to ER–PM Junctions and Regulates Store-Operated Ca^2+^ Entry. J. Cell Biol..

[171] Balla T., Kim Y.J., Alvarez-Prats A., Pemberton J. (2019). Lipid Dynamics at Contact Sites between the Endoplasmic Reticulum and Other Organelles. Annu. Rev. Cell Dev. Biol..

[172] Dell’Acqua M.L., Faux M.C., Thorburn J., Thorburn A., Scott J.D. (1998). Membrane-Targeting Sequences on AKAP79 Bind Phosphatidylinositol-4, 5-Bisphosphate. EMBO J..

[173] Blunsom N.J., Cockcroft S. (2020). CDP-Diacylglycerol Synthases (CDS): Gateway to Phosphatidylinositol and Cardiolipin Synthesis. Front. Cell Dev. Biol..

[174] Ma Q., Gabelli S.B., Raben D.M. (2019). Diacylglycerol Kinases: Relationship to Other Lipid Kinases. Adv. Biol. Regul..

[175] Collado J., Kalemanov M., Campelo F., Bourgoint C., Thomas F., Loewith R., Martínez-Sánchez A., Baumeister W., Stefan C.J., Fernández-Busnadiego R. (2019). Tricalbin-Mediated Contact Sites Control ER Curvature to Maintain Plasma Membrane Integrity. Dev. Cell.

[176] Besprozvannaya M., Dickson E., Li H., Ginburg K.S., Bers D.M., Auwerx J., Nunnari J. (2018). GRAM Domain Proteins Specialize Functionally Distinct ER-PM Contact Sites in Human Cells. eLife.

[177] Sandhu J., Li S., Fairall L., Pfisterer S.G., Gurnett J.E., Xiao X., Weston T.A., Vashi D., Ferrari A., Orozco J.L. (2018). Aster Proteins Facilitate Nonvesicular Plasma Membrane to ER Cholesterol Transport in Mammalian Cells. Cell.

[178] Johnson B., Leek A.N., Solé L., Maverick E.E., Levine T.P., Tamkun M.M. (2018). Kv2 Potassium Channels Form Endoplasmic Reticulum/Plasma Membrane Junctions via Interaction with VAPA and VAPB. Proc. Natl. Acad. Sci. USA.

[179] Johnson B., Leek A.N., Tamkun M.M. (2019). Kv2 Channels Create Endoplasmic Reticulum/Plasma Membrane Junctions: A Brief History of Kv2 Channel Subcellular Localization. Channels.

[180] Amarilio R., Ramachandran S., Sabanay H., Lev S. (2005). Differential Regulation of Endoplasmic Reticulum Structure through VAP-Nir Protein Interaction. J. Biol. Chem..

[181] Kim Y.J., Guzman-Hernandez M.-L., Wisniewski E., Balla T. (2015). Phosphatidylinositol-Phosphatidic Acid Exchange by Nir2 at ER-PM Contact Sites Maintains Phosphoinositide Signaling Competence. Dev. Cell.

[182] Kim S., Kedan A., Marom M., Gavert N., Keinan O., Selitrennik M., Laufman O., Lev S. (2013). The Phosphatidylinositol-Transfer Protein Nir2 Binds Phosphatidic Acid and Positively Regulates Phosphoinositide Signalling. EMBO Rep..

[183] Vig M., Peinelt C., Beck A., Koomoa D.L., Rabah D., Koblan-Huberson M., Kraft S., Turner H., Fleig A., Penner R. (2006). CRACM1 Is a Plasma Membrane Protein Essential for Store-Operated Ca^2+^ Entry. Science.

[184] Peinelt C., Vig M., Koomoa D.L., Beck A., Nadler M.J.S., Koblan-Huberson M., Lis A., Fleig A., Penner R., Kinet J.-P. (2006). Amplification of CRAC Current by STIM1 and CRACM1 (Orai1). Nat. Cell Biol..

[185] Soboloff J., Spassova M.A., Tang X.D., Hewavitharana T., Xu W., Gill D.L. (2006). Orai1 and STIM Reconstitute Store-Operated Calcium Channel Function*. J. Biol. Chem..

[186] Mercer J.C., DeHaven W.I., Smyth J.T., Wedel B., Boyles R.R., Bird G.S., Putney J.W. (2006). Large store-operated calcium-selective currents due to co-expression of orai1 or orai2 with the intracellular calcium sensor, STIM1. J. Biol. Chem..

[187] Wyles J.P., McMaster C.R., Ridgway N.D. (2002). Vesicle-Associated Membrane Protein-Associated Protein-A (VAP-A) Interacts with the Oxysterol-Binding Protein to Modify Export from the Endoplasmic Reticulum. J. Biol. Chem..

[188] Antonny B., Bigay J., Mesmin B. (2018). The Oxysterol-Binding Protein Cycle: Burning off PI(4)P to Transport Cholesterol. Annu. Rev. Biochem..

[189] Lehto M., Hynynen R., Karjalainen K., Kuismanen E., Hyvärinen K., Olkkonen V.M. (2005). Targeting of OSBP-Related Protein 3 (ORP3) to Endoplasmic Reticulum and Plasma Membrane Is Controlled by Multiple Determinants. Exp. Cell Res..

[190] Gulyás G., Sohn M., Kim Y.J., Várnai P., Balla T. (2020). ORP3 Phosphorylation Regulates Phosphatidylinositol 4-Phosphate and Ca^2+^ Dynamics at Plasma Membrane–ER Contact Sites. J. Cell Sci..

[191] Du X., Kumar J., Ferguson C., Schulz T.A., Ong Y.S., Hong W., Prinz W.A., Parton R.G., Brown A.J., Yang H. (2011). A Role for Oxysterol-Binding Protein-Related Protein 5 in Endosomal Cholesterol Trafficking. J. Cell Biol..

[192] Yan D., Mäyränpää M.I., Wong J., Perttilä J., Lehto M., Jauhiainen M., Kovanen P.T., Ehnholm C., Brown A.J., Olkkonen V.M. (2008). OSBP-Related Protein 8 (ORP8) Suppresses ABCA1 Expression and Cholesterol Efflux from Macrophages. J. Biol. Chem..

[193] Maeda K., Anand K., Chiapparino A., Kumar A., Poletto M., Kaksonen M., Gavin A.-C. (2013). Interactome Map Uncovers Phosphatidylserine Transport by Oxysterol-Binding Proteins. Nature.

[194] Ghai R., Du X., Wang H., Dong J., Ferguson C., Brown A.J., Parton R.G., Wu J.-W., Yang H. (2017). ORP5 and ORP8 Bind Phosphatidylinositol-4, 5-Biphosphate (PtdIns(4,5)P2) and Regulate Its Level at the Plasma Membrane. Nat. Commun..

[195] Sohn M., Korzeniowski M., Zewe J.P., Wills R.C., Hammond G.R.V., Humpolickova J., Vrzal L., Chalupska D., Veverka V., Fairn G.D. (2018). PI(4,5)P2 Controls Plasma Membrane PI4P and PS Levels via ORP5/8 Recruitment to ER-PM Contact Sites. J. Cell Biol..

[196] Heilmeyer L.M.G., Vereb G., Vereb G., Kakuk A., Szivák I. (2003). Mammalian Phosphatidylinositol 4-Kinases. IUBMB Life.

[197] Kanaho Y., Kobayashi-Nakano A., Yokozeki T. (2007). The Phosphoinositide Kinase PIP5K That Produces the Versatile Signaling Phospholipid PI4,5P(2). Biol. Pharm. Bull..

[198] Blunsom N.J., Cockcroft S. (2020). Phosphatidylinositol Synthesis at the Endoplasmic Reticulum. Biochim. Et Biophys. Acta (BBA)-Mol. Cell Biol. Lipids.

[199] Lefkimmiatis K., Zaccolo M. (2014). CAMP Signaling in Subcellular Compartments. Pharmacol. Ther..

[200] Kadamur G., Ross E.M. (2013). Mammalian Phospholipase C. Annu. Rev. Physiol..

[201] Del Bel L.M., Brill J.A. (2018). Sac1, a Lipid Phosphatase at the Interface of Vesicular and Nonvesicular Transport. Traffic.

[202] Maléth J., Choi S., Muallem S., Ahuja M. (2014). Translocation between PI(4,5)P2-Poor and PI(4,5)P2-Rich Microdomains during Store Depletion Determines STIM1 Conformation and Orai1 Gating. Nat. Commun..

[203] Zhang J., Kong C., Xie H., McPherson P.S., Grinstein S., Trimble W.S. (1999). Phosphatidylinositol Polyphosphate Binding to the Mammalian Septin H5 Is Modulated by GTP. Curr. Biol..

[204] Vandecaetsbeek I., Vangheluwe P., Raeymaekers L., Wuytack F., Vanoevelen J. (2011). The Ca^2+^ Pumps of the Endoplasmic Reticulum and Golgi Apparatus. Cold Spring Harb. Perspect. Biol..

[205] Verboomen H., Mertens L., Eggermont J., Wuytack F., Van Den Bosch L. (1995). Modulation of SERCA2 Activity: Regulated Splicing and Interaction with Phospholamban. Biosci. Rep..

[206] Liou J., Kim M.L., Do Heo W., Jones J.T., Myers J.W., Ferrell J.E., Meyer T. (2005). STIM Is a Ca^2+^ Sensor Essential for Ca^2+^-Store-Depletion-Triggered Ca^2+^ Influx. Curr. Biol..

[207] Roos J., DiGregorio P.J., Yeromin A.V., Ohlsen K., Lioudyno M., Zhang S., Safrina O., Kozak J.A., Wagner S.L., Cahalan M.D. (2005). STIM1, an Essential and Conserved Component of Store-Operated Ca^2+^ Channel Function. J. Cell Biol..

[208] Ercan E., Momburg F., Engel U., Temmerman K., Nickel W., Seedorf M. (2009). A Conserved, Lipid-Mediated Sorting Mechanism of Yeast Ist2 and Mammalian STIM Proteins to the Peripheral ER. Traffic.

[209] Wang Q.-C., Wang X., Tang T.-S. (2018). EB1 Traps STIM1 and Regulates Local Store-Operated Ca^2+^ Entry. J. Cell Biol..

[210] López J.J., Salido G.M., Pariente J.A., Rosado J.A. (2006). Interaction of STIM1 with Endogenously Expressed Human Canonical TRP1 upon Depletion of Intracellular Ca^2+^ Stores. J. Biol. Chem..

[211] Lees J.A., Messa M., Sun E.W., Wheeler H., Torta F., Wenk M.R., De Camilli P., Reinisch K.M. (2017). Lipid Transport by TMEM24 at ER-Plasma Membrane Contacts Regulates Pulsatile Insulin Secretion. Science.

[212] Sun E.W., Guillén-Samander A., Bian X., Wu Y., Cai Y., Messa M., De Camilli P. (2019). Lipid Transporter TMEM24/C2CD2L Is a Ca^2+^-Regulated Component of ER–Plasma Membrane Contacts in Mammalian Neurons. Proc. Natl. Acad. Sci. USA.

[213] Wes P.D., Chevesich J., Jeromin A., Rosenberg C., Stetten G., Montell C. (1995). TRPC1, a Human Homolog of a Drosophila Store-Operated Channel. Proc. Natl. Acad. Sci. USA.

[214] Zhu X., Chu P.B., Peyton M., Birnbaumer L. (1995). Molecular Cloning of a Widely Expressed Human Homologue for the Drosophila Trp Gene. FEBS Lett..

[215] Lee K.P., Choi S., Hong J.H., Ahuja M., Graham S., Ma R., So I., Shin D.M., Muallem S., Yuan J.P. (2014). Molecular Determinants Mediating Gating of Transient Receptor Potential Canonical (TRPC) Channels by Stromal Interaction Molecule 1 (STIM1). J. Biol. Chem..

[216] Murphy S.E., Levine T.P. (2016). VAP, a Versatile Access Point for the Endoplasmic Reticulum: Review and Analysis of FFAT-like Motifs in the VAPome. Biochim. Biophys. Acta.

[217] Yu H., Liu Y., Gulbranson D.R., Paine A., Rathore S.S., Shen J. (2016). Extended Synaptotagmins Are Ca^2+^-Dependent Lipid Transfer Proteins at Membrane Contact Sites. Proc. Natl. Acad. Sci. USA.

[218] Saheki Y., De Camilli P. (2017). The Extended-Synaptotagmins. Biochim. Biophys. Acta.

[219] Corradi V., Sejdiu B.I., Mesa-Galloso H., Abdizadeh H., Noskov S.Y., Marrink S.J., Tieleman D.P. (2019). Emerging Diversity in Lipid–Protein Interactions. Chem. Rev..

[220] Grouleff J., Irudayam S.J., Skeby K.K., Schiøtt B. (2015). The Influence of Cholesterol on Membrane Protein Structure, Function, and Dynamics Studied by Molecular Dynamics Simulations. Biochim. Et Biophys. Acta (BBA)-Biomembr..

[221] Muller M.P., Jiang T., Sun C., Lihan M., Pant S., Mahinthichaichan P., Trifan A., Tajkhorshid E. (2019). Characterization of Lipid-Protein Interactions and Lipid-Mediated Modulation of Membrane Protein Function Through Molecular Simulation. Chem. Rev..

[222] Bieberich E. (2018). Sphingolipids and Lipid Rafts: Novel Concepts and Methods of Analysis. Chem. Phys. Lipids.

[223] Lu S.M., Fairn G.D. (2018). Mesoscale Organization of Domains in the Plasma Membrane—beyond the Lipid Raft. Crit. Rev. Biochem. Mol. Biol..

[224] Rheinstädter M.C., Mouritsen O.G. (2013). Small-Scale Structure in Fluid Cholesterol–Lipid Bilayers. Curr. Opin. Colloid Interface Sci..

[225] Jardin I., Salido G.M., Rosado J.A. (2008). Role of Lipid Rafts in the Interaction between HTRPC1, Orai1 and STIM1. Channels.

[226] Galan C., Woodard G.E., Dionisio N., Salido G.M., Rosado J.A. (2010). Lipid Rafts Modulate the Activation but Not the Maintenance of Store-Operated Ca^2+^ Entry. Biochim. Et Biophys. Acta (BBA)-Mol. Cell Res..

[227] Lockwich T.P., Liu X., Singh B.B., Jadlowiec J., Weiland S., Ambudkar I.S. (2000). Assembly of Trp1 in a Signaling Complex Associated with Caveolin-Scaffolding Lipid Raft Domains. J. Biol. Chem..

[228] Murata T., Lin M.I., Stan R.V., Bauer P.M., Yu J., Sessa W.C. (2007). Genetic Evidence Supporting Caveolae Microdomain Regulation of Calcium Entry in Endothelial Cells. J. Biol. Chem..

[229] Prakash Y.S., Thompson M.A., Vaa B., Matabdin I., Peterson T.E., He T., Pabelick C.M. (2007). Caveolins and Intracellular Calcium Regulation in Human Airway Smooth Muscle. Am. J. Physiol. Lung Cell. Mol. Physiol..

[230] Pani B., Liu X., Bollimuntha S., Cheng K.T., Niesman I.R., Zheng C., Achen V.R., Patel H.H., Ambudkar I.S., Singh B.B. (2013). Impairment of TRPC1–STIM1 Channel Assembly and AQP5 Translocation Compromise Agonist-Stimulated Fluid Secretion in Mice Lacking Caveolin1. J. Cell Sci..

[231] Rathor N., Chung H.K., Wang S.R., Wang J., Turner D.J., Rao J.N. (2014). Caveolin-1 Enhances Rapid Mucosal Restitution by Activating TRPC1-mediated Ca^2+^ Signaling. Physiol. Rep..

[232] Alicia S., Angélica Z., Carlos S., Alfonso S., Vaca L. (2008). STIM1 Converts TRPC1 from a Receptor-Operated to a Store-Operated Channel: Moving TRPC1 in and out of Lipid Rafts. Cell Calcium.

[233] Pani B., Ong H.L., Liu X., Rauser K., Ambudkar I.S., Singh B.B. (2008). Lipid Rafts Determine Clustering of STIM1 in Endoplasmic Reticulum-Plasma Membrane Junctions and Regulation of Store-Operated Ca^2+^ Entry (SOCE). J. Biol. Chem..

[234] Pani B., Ong H.L., Brazer S.W., Liu X., Rauser K., Singh B.B., Ambudkar I.S. (2009). Activation of TRPC1 by STIM1 in ER-PM Microdomains Involves Release of the Channel from Its Scaffold Caveolin-1. Proc. Natl. Acad. Sci. USA.

[235] Sathish V., Abcejo A.J., Thompson M.A., Sieck G.C., Prakash Y.S., Pabelick C.M. (2012). Caveolin-1 Regulation of Store-Operated Ca^2+^ Influx in Human Airway Smooth Muscle. Eur. Respir. J..

[236] Yu F., Sun L., Machaca K. (2010). Constitutive Recycling of the Store-Operated Ca^2+^ Channel Orai1 and Its Internalization during Meiosis. J. Cell Biol..

[237] Fridolfsson H.N., Roth D.M., Insel P.A., Patel H.H. (2014). Regulation of Intracellular Signaling and Function by Caveolin. FASEB J..

[238] Yeh Y.-C., Parekh A.B. (2015). Distinct Structural Domains of Caveolin-1 Independently Regulate Ca^2+^ Release-Activated Ca^2+^ Channels and Ca^2+^ Microdomain-Dependent Gene Expression. Mol. Cell Biol..

[239] Gwozdz T., Dutko-Gwozdz J., Schafer C., Bolotina V.M. (2012). Overexpression of Orai1 and STIM1 Proteins Alters Regulation of Store-Operated Ca^2+^ Entry by Endogenous Mediators. J. Biol. Chem..

[240] Dionisio N., Galán C., Jardín I., Salido G.M., Rosado J.A. (2011). Lipid Rafts Are Essential for the Regulation of SOCE by Plasma Membrane Resident STIM1 in Human Platelets. Biochim. Et Biophys. Acta (BBA)-Mol. Cell Res..

[241] Derler I., Jardin I., Stathopulos P.B., Muik M., Fahrner M., Zayats V., Pandey S.K., Poteser M., Lackner B., Absolonova M. (2016). Cholesterol Modulates Orai1 Channel Function. Sci. Signal..

[242] Bóhorquez-Hernández A., Gratton E., Pacheco J., Asanov A., Vaca L. (2017). Cholesterol Modulates the Cellular Localization of Orai1 Channels and Its Disposition among Membrane Domains. Biochim. Biophys. Acta.

[243] Pacheco J., Dominguez L., Bohórquez-Hernández A., Asanov A., Vaca L. (2016). A Cholesterol-Binding Domain in STIM1 Modulates STIM1-Orai1 Physical and Functional Interactions. Sci. Rep..

[244] Doktorova M., Symons J., Levental I. (2020). Structural and Functional Consequences of Reversible Lipid Asymmetry in Living Membranes. Nat. Chem. Biol..

[245] Collins S.R., Meyer T. (2011). Evolutionary Origins of STIM1 and STIM2 within Ancient Ca^2+^ Signaling Systems. Trends Cell Biol..

[246] Heo W.D., Inoue T., Park W.S., Kim M.L., Park B.O., Wandless T.J., Meyer T. (2006). PI(3,4,5)P3 and PI(4,5)P2 Lipids Target Proteins with Polybasic Clusters to the Plasma Membrane. Science.

[247] Bhardwaj R., Müller H.-M., Nickel W., Seedorf M. (2013). Oligomerization and Ca^2+^/Calmodulin Control Binding of the ER Ca^2+^-Sensors STIM1 and STIM2 to Plasma Membrane Lipids. Biosci. Rep..

[248] Korzeniowski M.K., Popovic M.A., Szentpetery Z., Varnai P., Stojilkovic S.S., Balla T. (2009). Dependence of STIM1/Orai1-Mediated Calcium Entry on Plasma Membrane Phosphoinositides. J. Biol. Chem..

[249] Calloway N., Owens T., Corwith K., Rodgers W., Holowka D., Baird B. (2011). Stimulated Association of STIM1 and Orai1 Is Regulated by the Balance of PtdIns(4,5)P2 between Distinct Membrane Pools. J. Cell Sci..

[250] Imai Y., Itsuki K., Okamura Y., Inoue R., Mori M.X. (2012). A Self-Limiting Regulation of Vasoconstrictor-Activated TRPC3/C6/C7 Channels Coupled to PI(4,5)P2-Diacylglycerol Signalling. J. Physiol..

[251] Itsuki K., Imai Y., Hase H., Okamura Y., Inoue R., Mori M.X. (2014). PLC-Mediated PI(4,5)P2 Hydrolysis Regulates Activation and Inactivation of TRPC6/7 Channels. J. Gen. Physiol..

[252] Kim H., Jeon J.-P., Hong C., Kim J., Myeong J., Jeon J.-H., So I. (2013). An Essential Role of PI(4,5)P2 for Maintaining the Activity of the Transient Receptor Potential Canonical (TRPC)4β. Pflug. Arch. Eur. J. Physiol..

[253] Shi J., Birnbaumer L., Large W.A., Albert A.P. (2014). Myristoylated Alanine-Rich C Kinase Substrate Coordinates Native TRPC1 Channel Activation by Phosphatidylinositol 4,5-Bisphosphate and Protein Kinase C in Vascular Smooth Muscle. FASEB J..

[254] Bodnar D., Chung W.Y., Yang D., Hong J.H., Jha A., Muallem S., Groschner K., Graier W.F., Romanin C. (2017). STIM-TRP Pathways and Microdomain Organization: Ca^2+^ Influx Channels: The Orai-STIM1-TRPC Complexes. Store-Operated Ca^2+^ Entry (SOCE) Pathways: Emerging Signaling Concepts in Human (Patho)physiology.

[255] Ando H., Mizutani A., Matsu-ura T., Mikoshiba K. (2003). IRBIT, a Novel Inositol 1,4,5-Trisphosphate (IP3) Receptor-Binding Protein, Is Released from the IP3 Receptor upon IP3 Binding to the Receptor. J. Biol. Chem..

[256] Cooper D.M.F. (2015). Store-Operated Ca^2+^-Entry and Adenylyl Cyclase. Cell Calcium.

[257] Pagano M., Clynes M.A., Masada N., Ciruela A., Ayling L.-J., Wachten S., Cooper D.M.F. (2009). Insights into the Residence in Lipid Rafts of Adenylyl Cyclase AC8 and Its Regulation by Capacitative Calcium Entry. Am. J. Physiol. Cell Physiol..

[258] Tabbasum V.G., Cooper D.M.F. (2019). Structural and Functional Determinants of AC8 Trafficking, Targeting and Responsiveness in Lipid Raft Microdomains. J. Membr. Biol..

[259] Ayling L.J., Briddon S.J., Halls M.L., Hammond G.R.V., Vaca L., Pacheco J., Hill S.J., Cooper D.M.F. (2012). Adenylyl Cyclase AC8 Directly Controls Its Micro-Environment by Recruiting the Actin Cytoskeleton in a Cholesterol-Rich Milieu. J. Cell Sci..

[260] Delint-Ramirez I., Willoughby D., Hammond G.R.V., Hammond G.V.R., Ayling L.J., Cooper D.M.F. (2011). Palmitoylation Targets AKAP79 Protein to Lipid Rafts and Promotes Its Regulation of Calcium-Sensitive Adenylyl Cyclase Type 8. J. Biol. Chem..

[261] Cornelius F., Habeck M., Kanai R., Toyoshima C., Karlish S.J.D. (2015). General and Specific Lipid–Protein Interactions in Na,K-ATPase. Biochim. Et Biophys. Acta (BBA)-Biomembr..

[262] Denning E.J., Beckstein O. (2013). Influence of Lipids on Protein-Mediated Transmembrane Transport. Chem. Phys. Lipids.

[263] Locke D., Harris A.L. (2009). Connexin Channels and Phospholipids: Association and Modulation. BMC Biol..

[264] Shuttleworth T.J. (2017). Selective Activation of Distinct Orai Channels by STIM1. Cell Calcium.

[265] Hille B., Dickson E.J., Kruse M., Vivas O., Suh B.-C. (2015). Phosphoinositides Regulate Ion Channels. Biochim. Biophys. Acta.

[266] Aharonovitz O., Zaun H.C., Balla T., York J.D., Orlowski J., Grinstein S. (2000). Intracellular Ph Regulation by Na+/H+ Exchange Requires Phosphatidylinositol 4,5-Bisphosphate. J. Cell Biol..

[267] Hong J.H., Yang D., Shcheynikov N., Ohana E., Shin D.M., Muallem S. (2013). Convergence of IRBIT, Phosphatidylinositol (4,5) Bisphosphate, and WNK/SPAK Kinases in Regulation of the Na+-HCO3− Cotransporters Family. Proc. Natl. Acad. Sci. USA.

[268] Wu J., McNicholas C.M., Bevensee M.O. (2009). Phosphatidylinositol 4,5-Bisphosphate (PIP2) Stimulates the Electrogenic Na/HCO3 Cotransporter NBCe1-A Expressed in Xenopus Oocytes. Proc. Natl. Acad. Sci. USA.

[269] Lopreiato R., Giacomello M., Carafoli E. (2014). The Plasma Membrane Calcium Pump: New Ways to Look at an Old Enzyme. J. Biol. Chem..

[270] Hansen S.B., Tao X., MacKinnon R. (2011). Structural Basis of PIP2 Activation of the Classical Inward Rectifier K+ Channel Kir2.2. Nature.

[271] Whorton M.R., MacKinnon R. (2011). Crystal Structure of the Mammalian GIRK2 K+ Channel and Gating Regulation by G Proteins, PIP2, and Sodium. Cell.

[272] Taylor K.C., Sanders C.R. (2017). Regulation of KCNQ/Kv7 Family Voltage-Gated K+ Channels by Lipids. Biochim. Biophys. Acta.

[273] Katona M., Vizvári E., Németh L., Facskó A., Venglovecz V., Rakonczay Z., Hegyi P., Tóth-Molnár E. (2014). Experimental Evidence of Fluid Secretion of Rabbit Lacrimal Gland Duct Epithelium. Investig. Ophthalmol. Vis. Sci..

[274] Vizvári E., Katona M., Orvos P., Berczeli O., Facskó A., Rárosi F., Venglovecz V., Rakonczay Z., Hegyi P., Ding C. (2016). Characterization of Na+-K+-2Cl- Cotransporter Activity in Rabbit Lacrimal Gland Duct Cells. Investig. Ophthalmol. Vis. Sci..

[275] Berczeli O., Vizvári E., Katona M., Török D., Szalay L., Rárosi F., Németh I., Rakonczay Z., Hegyi P., Ding C. (2018). Novel Insight into the Role of CFTR in Lacrimal Gland Duct Function in Mice. Investig. Ophthalmol. Vis. Sci..

[276] Szarka D., Elekes G., Berczeli O., Vizvári E., Szalay L., Ding C., Tálosi L., Tóth-Molnár E. (2020). Alpha-Adrenergic Agonists Stimulate Fluid Secretion in Lacrimal Gland Ducts. Investig. Ophthalmol. Vis. Sci..

[277] Lee M.G., Ohana E., Park H.W., Yang D., Muallem S. (2012). Molecular Mechanism of Pancreatic and Salivary Gland Fluid and HCO3 Secretion. Physiol. Rev..

[278] Hegyi P., Maléth J., Venglovecz V., Rakonczay Z. (2011). Pancreatic Ductal Bicarbonate Secretion: Challenge of the Acinar Acid Load. Front. Physiol..

